# Constructing Dual‐Atomic Fe─Fe Sites Nanozyme for Targeted Osteoarthritis Therapy through Mitigating Oxidative Stress and Cartilage Degeneration

**DOI:** 10.1002/advs.202508073

**Published:** 2025-10-20

**Authors:** Ting Ying, Qi Wang, Dejian Li, Yao Wang, Pengfang Zhang, Chengqing Yi, Rui Zhu

**Affiliations:** ^1^ Shanghai Yangzhi Rehabilitation Hospital (Shanghai Sunshine Rehabilitation Center) School of Medicine Tongji University Shanghai 200092 China; ^2^ Department of Orthopedics Shanghai Pudong Hospital Fudan University Pudong Medical Center Shanghai 201300 China; ^3^ Key Laboratory of Synthetic and Biological Colloids Ministry of Education School of Chemical and Material Engineering Jiangnan University Wuxi 214122 China; ^4^ Shandong Provincial Key Laboratory of Chemical Energy Storage and Novel Cell Technology Liaocheng University Liaocheng 252000 China

**Keywords:** dual‐atom sites, Fe‐based nanozyme, osteoarthritis, reactive oxygen species, targeted antioxidant therapy

## Abstract

Osteoarthritis progression is driven by excessive reactive oxygen species (ROS), which causes significant secondary damage to chondrocytes and articular cartilage. Herein, the concept of holding desired dual‐site nanozymes is proposed through developing Fe─Fe dimers on nitrogen‐doped porous carbon (Fe_2_‐NCs) to eliminate excessive ROS through the co‐adsorption mechanism of superoxide radical. The Fe_2_‐NCs present an enhanced ROS performance, effectively mimicking key antioxidant enzymes. Density functional theory calculations indicate that the synergistic effects of the Fe─Fe dimer can modulate oxygen adsorption configurations and accelerating O─O bond‐cleavage. In vitro and in vivo results show that Fe_2_‐NCs effectively mitigate ROS, protecting chondrocytes from oxidative stress‐induced apoptosis. The mitochondrial function can be strengthened over Fe_2_‐NCs by inhibiting NOX4 expression, restoring ATP levels, and normalizing COXIV expression. Additionally, Fe_2_‐NCs significantly downregulate the pro‐inflammatory mediator COX‐2, inhibit MMP‐13‐mediated cartilage degradation, and slow down the type II collagen (COL2) breakdown through the inhibition of NF‐κB signaling pathway.

## Graphical abstract

A novel bimetallic nanozyme (Fe_2_–NCs) with well‐defined Fe–Fe active sites is synthesized through a host–guest coordination strategy, which exhibits an exceptional therapeutic efficacy in osteoarthritis therapy. Experimental and theoretical analyses uncover the intrinsic catalytic mechanism at the atomic level, offering fundamental insights and design principles for the future development of dual‐atom nanomaterials in biomedical applications.

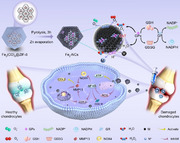

## 1 Introduction

Osteoarthritis (OA) is a prevalent degenerative joint disorder.^[^ ^1^ ^]^ Growing evidence has highlighted the critical involvement of reactive oxygen species (ROS) in the pathophysiology of OA.^[^ ^2^, ^3^, ^4^, ^5^ ^]^ In the OA pathological microevironment, excessive ROS (e.g., •OH, H_2_O_2_, O_2_•^−^) act as pivotal drivers of disease progression.^[^ ^6^ ^]^ The excessive accumulation of ROS can directly damage key cellular structures, while disturbing the redox homeostasis within mitochondria, activating critical signaling pathways, thereby amplifying the release of proinflammatory cytokines and exacerbating chondrocyte apoptosis.^[^ ^6^, ^7^, ^8^, ^9^, ^10^ ^]^ By doing so, this cascade culminates in catastrophic degradation of cartilage extracellular matrix (ECM) components—primarily collagen II and aggrecan—establishing a self‐perpetuating ROS‐inflammation‐ECM destruction cycle. Antioxidant enzymes such as superoxide dismutase (SOD), catalase (CAT), and glutathione peroxidase (GSH‐Px) have recently garnered significant attention due to their catalytic reactions within cells to remove ROS, thereby regulating the redox state and protecting cells from oxidative damage.^[^ ^11^, ^12^ ^]^ However, the low stability of these natural enzymes under pathological conditions limited their practical applications.^[^ ^13^, ^14^ ^]^ Consequently, there is a pressing need to enhance the stability of these antioxidant enzymes to unlock their full potential for therapeutic use in regenerative medicine and other clinical applications. Studies highlighted the exceptional catalytic performance of single‐atom NCs, which capitalize on the utilization of single metal atoms and the strong interactions between metal centers and supports, conferring a remarkable catalytic activity and selectivity of ROS removal.^[^ ^15^, ^16^, ^17^, ^18^ ^]^ Despite their promising capabilities in simulating the functions of natural oxidases, single‐atom NCs face significant challenges in replicating the intricate multinuclear structures found in natural enzymes, which hinders the effective adsorption and desorption of reaction intermediates during the catalytic process, ultimately reducing the efficiency of ROS catalysis.^[^ ^19^, ^20^ ^]^

To overcome this limitation, atomically dispersed dual‐atom NCs featuring two adjacent metal atom sites have emerged as a promising solution, which more closely resemble the multi‐metal active centers commonly in natural oxidases.^[^ ^21^, ^22^, ^23^ ^]^ In specific, atomically dispersed dual‐atom NCs can provide the highest atom utilization and thus offering the most active sites for biochemical reactions.^[^ ^21^, ^24^, ^25^ ^]^ Furthermore, with their well‐defined atomic‐level dispersion, these materials provide great targeting property for achieving high selectivity through promoting the adsorption/desorption of reaction intermediates and the breaking of O─O bonds.^[^ ^26^, ^27^ ^]^ Thus, designing and constructing a desired dual‐atom NCs with precise diatomic structures and understanding the deep mechanisms of catalytic ROS removal is necessary for next‐generation biomimetic enzyme. In addition, the natural antioxidant defense system comprises a complex network of multiple antioxidant enzymes working synergistically to protect cells from oxidative stress.^[^ ^13^, ^28^ ^]^ Notably, the reported nanozymes emulating these systems often encounter inherent limitations in mimicking the multiple‐antioxidant activities.^[^ ^29^ ^]^ Therefore, developing a dual‐atom NCs platform that accurately constructs homonuclear metal sites and can simulate multiple enzyme activities will better mimic complex intracellular antioxidant defense systems and provide new ideas for OA treatment.

The bimetallic Fe─Fe site exhibits excellent ROS scavenging ability due to its unique and highly synergistic effect. This synergistic effect helps to reduce the dissociation energy barrier of ROS and stabilize reactive oxygen species intermediates, thus significantly outperforming single‐atom or heteronuclear metal sites. This mechanism endows the diatomic Fe─Fe site with greater application value in clearing ROS and intervening in the OA pathological process. In addition, homonuclear Fe─Fe dimers exhibit excellent acid resistance and structural stability under the slightly acidic conditions of OA. In comparison, heterogeneous metal pairs (such as Fe─Cu) are prone to electronic mismatch, leading to structural disintegration of the active center. The most crucial aspect is that the diatomic Fe─Fe site highly mimics natural peroxidase in both structure and function, preserving its catalytic activity motif intact. This structural simulation helps to more effectively eliminate ROS, inhibit pro‐inflammatory signaling cascades, thereby synergistically maintaining chondrocyte vitality and slowing down cartilage matrix degradation, providing a strong foundation for OA intervention.

**Scheme 1:**
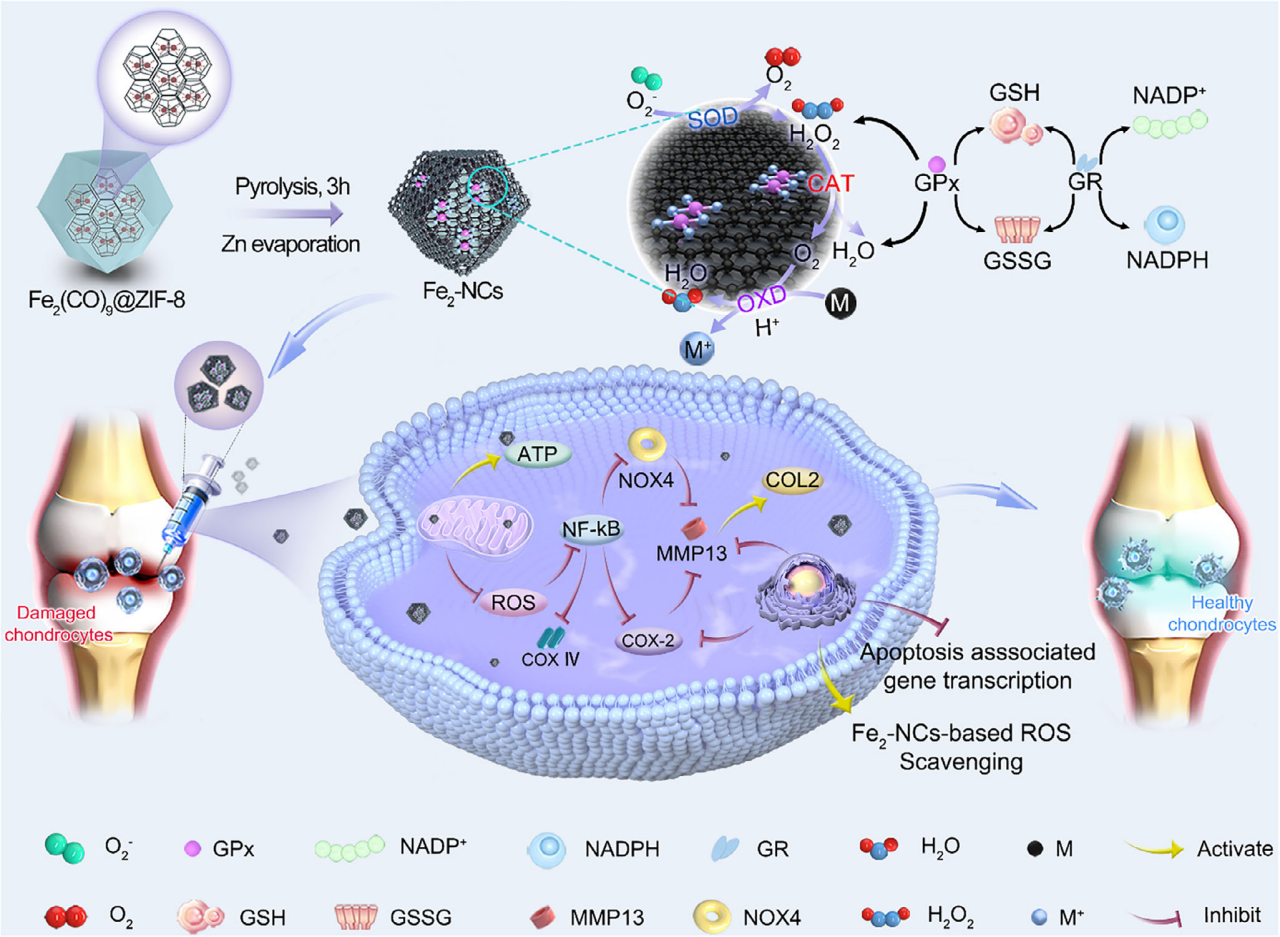
Schematic synthesis and mechanism illustration of Fe_2_‐NCs, which perform as SOD, CAT, and GSH‐Px to sequentially scavenge ROS and GSH for mitigating OA.

Herein, a nitrogen‐doped porous carbon anchored dual‐atom iron nanozyme catalysts (Fe_2_‐NCs) with a Fe─Fe dimer coordination was developed using a “host‐guest” strategy. As illustrated in **Scheme** **1**, the prepared Fe_2_‐NCs exhibit antioxidant enzyme‐like activities, including SOD, CAT, OXD, and GSH‐Px, effectively mimicking the natural antioxidant system. Compared to single‐atom iron nanozyme catalysts (Fe_1_‐NCs), Fe_2_‐NCs show superior catalytic performance, as supported by density functional theory (DFT) calculations, which suggests that the synergistic effect of the Fe─Fe dimer structure enhances oxygen adsorption configuration, prolongs the distance of O─O bonds and reduces the energy barrier of key transition states, thereby accelerating the cleavage of O─O bonds and enhancing the catalytic activity. In vitro experiments reveal that Fe_2_‐NCs protect chondrocytes from oxidative stress‐induced apoptosis by regulating ROS and reactive nitrogen species (RNS), and promoting O_2_ release. Furthermore, Fe_2_‐NCs restore mitochondrial function by inhibiting NOX4 expression, improving ATP production, and normalizing COXIV levels. In vivo OA model, Fe_2_‐NCs reduces the expression of pro‐inflammatory mediator COX‐2, inhibits MMP‐13 upregulation, and delays collagen II degradation, likely through the NF‐κB signaling pathway. This study provides a new theoretical framework and methodological approach for OA treatment, with significant clinical and scientific implications.

## 2 Results and Discussion

### 2.1 Preparation and Structural Characterization of Fe_2_‐NCs

**Figure 1:**
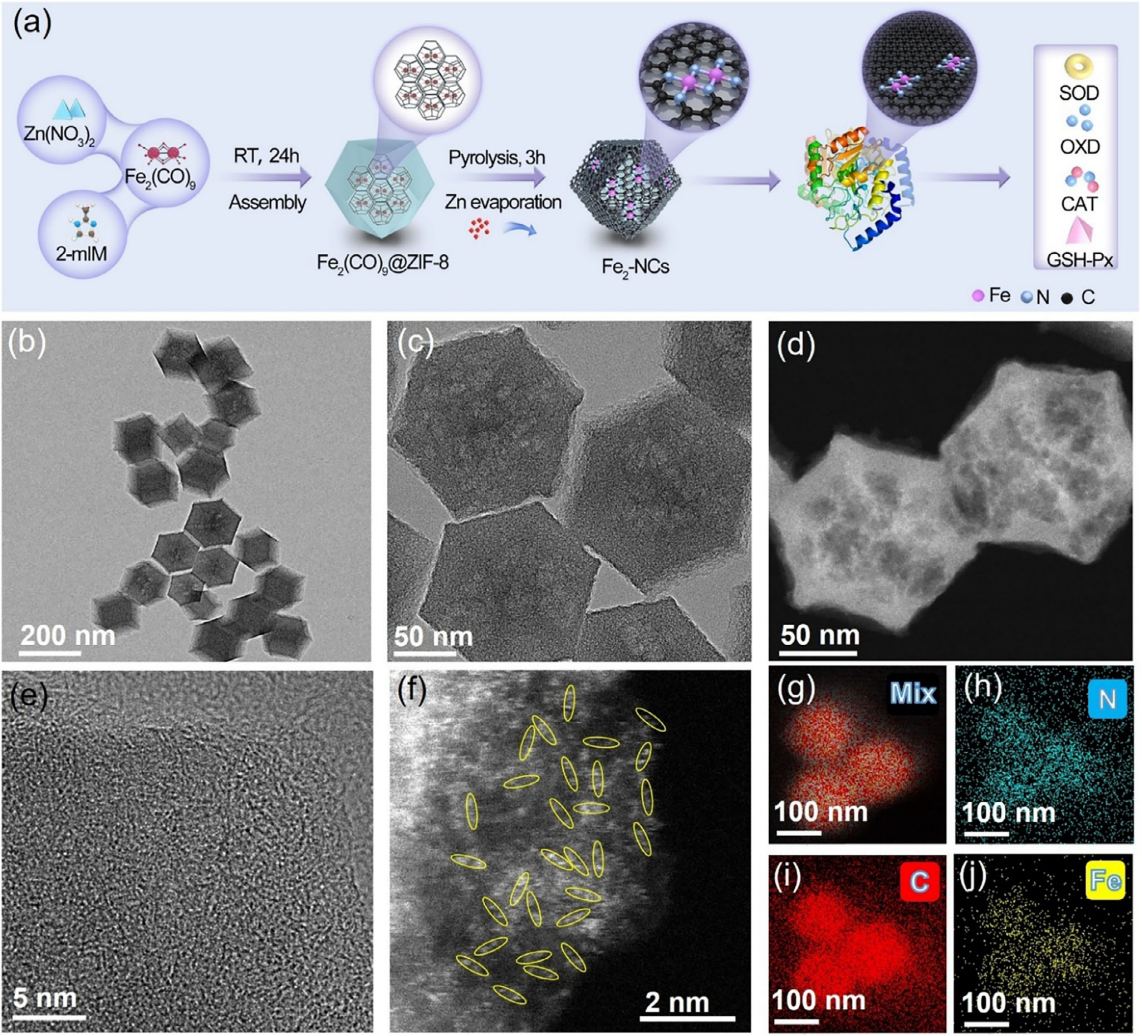
a) Schematic illustration of the synthesis of Fe_2_‐NCs. b,c) TEM images, d) STEM image, e) HRTEM image, and f) HAADF‐STEM image of Fe_2_‐NCs. g–j) AC‐HAADF‐STEM and corresponding EDX elemental mapping images of Fe_2_‐NCs.

To accurately design the atomically dispersed Fe_2_‐NCs, the “host‐guest” strategy was adopted as illustrated in **Figure** **1**a. First, the nonacarbonyldiiron (Fe_2_(CO)_9_) featuring an intrinsic Fe─Fe metallic bond (bond length: 2.52 Å), was judiciously incorporated into zeolitic imidazolate framework‐8 (ZIF‐8) via solution‐phase impregnation. The molecular‐scale encapsulation harnesses ZIF‐8 tetrahedral cavities (11.6 Å) interconnected through narrow apertures (3.4 Å), enabling size‐exclusive entrapment of Fe_2_(CO)_9_ guest molecules (with a molecular diameter of ≈9.5 Å), thereby forming Fe_2_(CO)_9_@ZIF‐8, which can ensure the atomic dispersion of Fe dimers, while preserving the rhombic dodecahedron morphology of ZIF‐8, as confirmed by unaltered crystallographic features by scanning electron microscopy (SEM) and transmission electron microscopy (TEM) analyses (Figures S1 and S2, Supporting Information). Second, the subsequent pyrolysis process derives Zn evaporation and Fe_2_ deposition on ligand nodes of the ZIF‐8 framework to obtain Fe_2_‐NCs. Morphological and structural characterizations of the Fe_2_‐NCs were systematically conducted by advanced electron microscopy techniques. As evidenced by SEM analysis in Figure S3 (Supporting Information), the synthesized material maintains a well‐defined polyhedral architecture with rhombic dodecahedral symmetry (average particle diameter: 110 nm), exhibiting no signs of morphological degradation. TEM investigations corroborate the preservation of rhombohedral framework integrity throughout the synthesis process (Figure 1b). High‐resolution TEM imaging (HRTEM, Figure 1c) reveals an intricate 3D mesoporous network within the nanocatalyst, further confirmed by scanning transmission electron microscopy (STEM) in Figure 1d. This hierarchical porous architecture substantially enhances active site accessibility through increased Fe‐N_x_ moiety exposure, while optimizing mass transfer kinetics during ROS catalytic cycles. HRTEM examination (Figure 1e) demonstrates the absence of metallic iron aggregates or crystalline nanoparticles, confirming atomic‐level dispersion of Fe species. Complementary high‐angle annular dark‐field STEM (HAADF‐STEM) mapping (Figure 1f) reveals a homogeneous distribution of diatomic Fe anchored on the nitrogen‐doped carbon matrix. Furthermore, the Zeta potentials of the NCs, Fe_1_‐NCs, and Fe_2_‐NCs were examined, and the corresponding potential values in Figure S4 (Supporting Information) exhibited that the surface electronegativity of the prepared samples were ≈26.23, 26.44, and 26.81 mV, respectively, indicating that the NCs, Fe_1_‐NCs, and Fe_2_‐NCs possess a moderately positive surface charge under these conditions. By doing so, the NCs, Fe_1_‐NCs, and Fe_2_‐NCs possessed moderate stability and bioavailability.

In addition, the corresponding power X‐ray diffraction (XRD) pattern in Figure S5 (Supporting Information) shows that no peaks corresponding to Fe_x_O or metallic Fe were observed, excluding the presence of large crystalline particles of Fe‐containing species.^[^ ^30^ ^]^ The selected‐area electron diffraction (SAED, Figure S6, Supporting Information) patterns also present an amorphous phase spectrogram. The energy‐dispersive spectroscopy (EDS) mapping demonstrates that C, N, and Fe elements are uniformly dispersed throughout the architecture of Fe_2_‐NCs (Figure 1g). As a comparison, the reference samples of single‐atomic Fe nanozyme (Fe_1_‐NCs) using ferric acetylacetonate (Fe(acac)_2_) as iron source and pure NCs carriers are obtained under similar synthesis methods. The distinct amorphous structure and morphology of Fe_1_‐NCs and NCs are clearly proved by XRD, SAED, and HAADF‐STEM techniques (Figures S7–S9, Supporting Information). Dynamic Light Scattering （DLS) results in Figure S10d (Supporting Information) further exhibited that the average of NCs, Fe_1_‐NCs and Fe_2_‐NCs was ≈113.9, 114.5 and 114.8 nm, which was slightly higher than a size distribution histogram (105.0, 105.2, and 105.1 nm, Figure S10a–c, Supporting Information), implying that the nanoparticles may present slightly larger sizes under physiological conditions due to the hydration shell, yet they remain adequately monodisperse, and there was no significant difference in the particle size of the as‐prepared nanozymes. The Fe contents in Fe_2_‐NCs and Fe_1_‐NCs are measured by inductively coupled plasma atomic emission spectroscopy (ICP‐AES) as 1.42 and 1.37 wt.%, respectively (Table S1, Supporting Information). N_2_ physisorption isotherms (Figure S11, Supporting Information) revealed Fe_2_‐NCs exhibits superior adsorption properties with a Brunauer–Emmett–Teller (BET) surface area of 808.04 m^2^g^−1^, significantly exceeding Fe_1_‐NCs (640.21 m^2^g^−1^) and NCs (407.89 m^2^g^−1^), which enhanced surface accessibility, critical for catalytic performance optimization, originating from nitrogen vacancy formation and anisotropic contraction of the carbon framework during pyrolysis.^[^ ^31^ ^]^ Raman spectroscopic analysis further confirmed structural evolution, displaying characteristic D‐band (1355 cm^−1^) and G‐band (1572 cm^−1^) intensities (Figure S12, Supporting Information). The elevated ID/IG ratio (1.263) in Fe_2_‐NCs vs Fe_1_‐NCs (1.128) and NCs (1.090) demonstrates synergistic coupling between adjacent Fe─Fe sites promotes defect engineering within the graphitic matrix, which facilitates the enhanced *π*‐electron delocalization, further maintaining sufficient graphitization for efficient charge transfer kinetics.^[^ ^32^ ^]^

**Figure 2:**
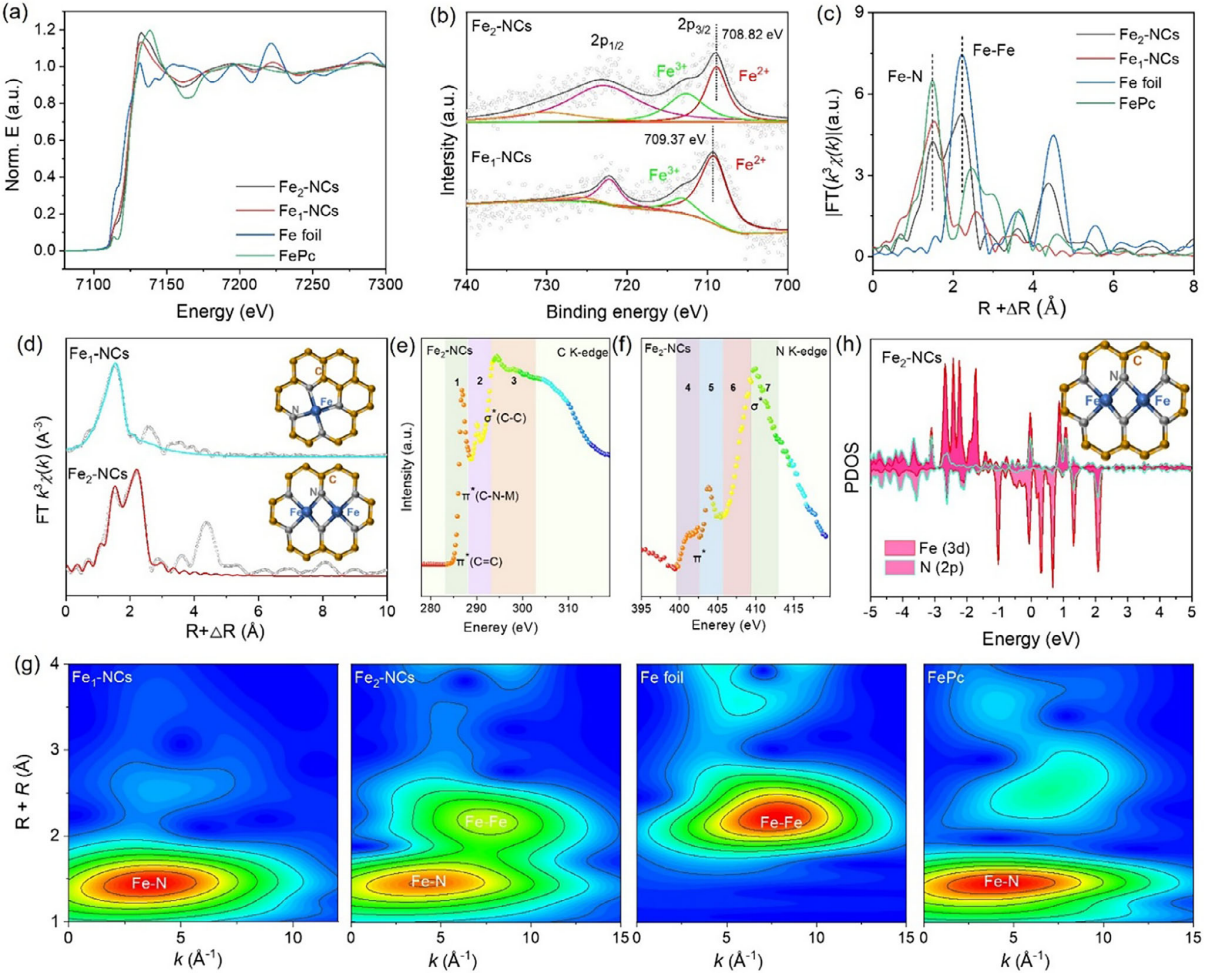
Multimodal spectroscopic characterization of Fe coordination environments. a) Fe K‐edge XANES profiles for Fe_2_‐NCs, Fe_1_‐NCs, Fe foil, Fe_2_O_3_), b) High‐resolution Fe 2p XPS spectra deconvoluted into 2p_3/2_ and 2p_1/2_ spin orbit components, c) FT k^3^‐weighted χ(k)‐function of EXAFS spectra comparing Fe─Fe/Fe─N coordination shells at Fe K‐edge of Fe_2_‐NCs and Fe_1_‐NCs. d) EXAFS oscillation fitting in R‐space with theoretical models (inset: optimized Fe_2_N_6_ and Fe_1_N_4_ configurations). e,f) C and N K‐edge XANES spectra revealing C═C π* (285.4 eV) and pyridinic N (398.6 eV) bonding states. g) WT for FT k^3^‐weighted χ(k)‐function of the EXAFS spectra of Fe_1_‐NCs and Fe_2_‐NCs. h) PDOS analysis of Fe‐3d, C‐2p, and N‐2p orbitals, highlighting d‐band center downshift (−1.7 eV vs Fermi level) in Fe_2_‐NCs.

The electronic configuration and atomic coordination of Fe species in Fe_2_‐NCs were systematically elucidated through synchrotron‐based X‐ray absorption spectroscopy (XAS) and X‐ray photoelectron spectroscopy (XPS) analyses.^[^ ^25^, ^33^ ^]^ X‐ray absorption near‐edge structure (XANES) profiles demonstrate the Fe K‐edge absorption thresholds for Fe_2_‐NCs and Fe_1_‐NCs reside between Fe foil and Fe_2_O_3_ (**Figure** **2**a), confirming an intermediate Fe species oxidation state (with an average value is ≈+2) through linear combination fitting. This valence state assignment is corroborated by high‐resolution Fe 2p XPS spectra (Figure 2b), where the Fe_2_‐NCs exhibit a 0.3 eV negative shift in 2p_3/2_ binding energy relative to Fe_1_‐NCs (710.8 vs 711.1 eV), indicative of enhanced electron delocalization within Fe─Fe dimers compared to isolated Fe‐N_x_ moieties, which is in favor of transforming ROS species. Extended X‐ray absorption fine structure (EXAFS) analysis reveals distinct coordination information (Figure 2c). The primary Fourier transform peak at 1.48 Å corresponds to Fe‐N first‐shell coordination (CN = 4.1 ± 0.2 for Fe_1_‐NCs; CN = 5.3 ± 0.9 for Fe_2_‐NCs). Notably, Fe_2_‐NCs exhibit a secondary scattering path at 2.49 Å (R‐space), characteristic of Fe─Fe metallic bonding, confirming atomic‐scale Fe_2_ dimer formation. Quantitative EXAFS fitting further verifies the co‐existence of Fe‐N_4_ (77%) and Fe─Fe (23%) coordination environment in Fe_2_‐NCs (Figure 2d; Table S2, Supporting Information). DFT modeling identifies the thermodynamically stable configuration of Fe─Fe pairs embedded in graphitic matrices (formation energy: −3.2 eV atom^−1^), where adjacent Fe atoms adopt bridge‐bonding geometry with 2.18 Å interatomic distance (Figure S13, Supporting Information). This unique diatomic architecture facilitates synergistic electron transfer, as evidenced by Bader charge analysis, which optimizes ROS transformation kinetics through enhanced d‐orbital hybridization (Figure 2d).

To elucidate the atomic coordination architecture of Fe sites in Fe_2_‐NCs, the synchrotron‐based soft X ray absorption near‐edge structure (XANES) and XPS were systematically employed. The C K‐edge XANES spectrum (Figure 2e) displays three distinct resonances at 286.9 eV (π*C═C), 289.9 eV (π*C─N─M), and 294.2 eV (σ*C─C), corresponding to transitions from C 1s to antibonding orbitals.^[^ ^34^ ^]^ Complementary XPS analysis corroborates these assignments through deconvolution of C 1s peaks at 283.5 eV (graphitic sp^2^ C═C), 284.8 eV (C─N coordination), and 287.1 eV (C─C σ bonds), respectively (Figure S14, Supporting Information). N K‐edge XANES reveals four characteristic excitations: pyridinic N (Peak 4, 398.5 eV), Fe─N coordination (Peak 5, 400.1 eV), pyrrolic N (Peak 6, 402.8 eV), and graphitic N (Peak 7, 405.3 eV) (Figure 2f; Table S3, Supporting Information). High‐resolution N 1s XPS quantifies nitrogen speciation with binding energies at 399.2 eV (Fe‐N_x_), 400.4 eV (pyrrolic N), 401.3 eV (pyridinic N), and 403.0 eV (graphitic N), confirming successful preservation of Fe‐N_x_ motifs during pyrolysis (Figure S15, Supporting Information).^[^ ^25^, ^35^ ^]^ Wavelet transform EXAFS analysis resolves two prominent scattering paths in Fe_2_‐NCs: a primary Fe─N coordination shell at 1.5 Å and a secondary Fe─Fe metallic bond at 2.3 Å, unambiguously confirming atomic Fe_2_ dimerization (Figure 2g).

The electronic configuration evolution within Fe_2_‐NCs was rigorously investigated through DFT simulations. As evidenced by the projected density of states (PDOS) analysis in Figure 2h, Fe_2_‐NCs exhibit enhanced d‐p orbital hybridization between adjacent Fe sites compared to Fe_1_‐NCs (Figure S16, Supporting Information), manifested by an obvious downshift of the d‐band center. 3D charge density difference mapping (Figure S17, Supporting Information) demonstrates significant electron redistribution around Fe_2_ dimers, characterized by an electron accumulation region at the Fe─Fe interatomic axis and depletion zones at Fe─N coordination sites. This asymmetric charge distribution establishes an optimized electronic environment for ROS activation, as quantified by enhanced adsorption energies. Electron localization function (ELF) analysis (Figure S18, Supporting Information) reveals a delocalization index within Fe_2_‐NCs, which is higher than that in Fe_1_‐NCs, indicating strengthened metallic character between Fe atoms. The interatomic ELF maxima along the Fe─Fe direction suggests partial covalent bonding, while maintaining ionic Fe─N interactions.

### 2.2 Enzyme‐Like Activity Evaluation and Theoretical Analysis

**Figure 3:**
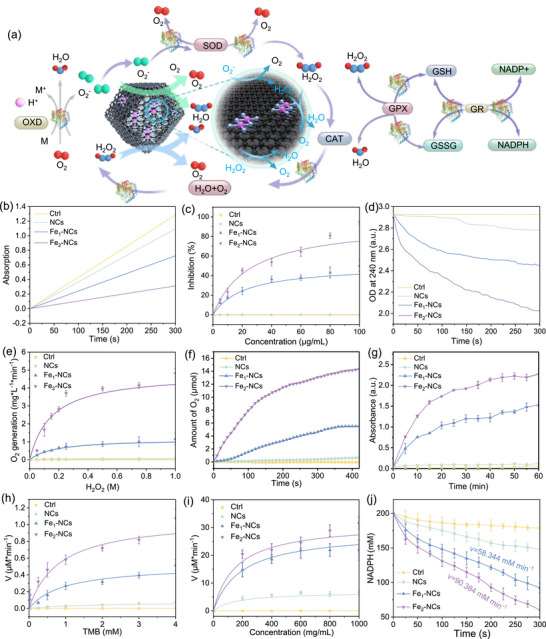
Multi‐enzyme catalytic cascade of Fe_2_‐NCs nanozymes. a) Bioinspired catalytic defense mechanism: Nanozyme‐mediated sequential ROS scavenging mimicking SOD, CAT, OXD, and GPx enzymatic cascades. b) SOD‐mimetic kinetics: Temporal reduction of WST‐8‐formazan complex absorbance at 450 nm (50 µg mL^−1^ nanozymes, 0.1 mm xanthine/xanthine oxidase O_2_^‐^· generation system). c) Dose‐dependent O_2_^‐^· radical scavenging efficiency. d) CAT‐like H_2_O_2_ decomposition kinetics monitored at 240 nm (0.5 mm H_2_O_2_, 25 °C). e) Michaelis‐Menten analysis of CAT‐mimetic activity. f) Dioxygen evolution profiles via H_2_O_2_ disproportionation (0.1 M H_2_O_2_, 20 µg mL^−1^ nanozymes). g) OXD‐mimetic TMB oxidation kinetics. h) Steady‐state OXD kinetics. i) Plots of initial rates (V_0_) at different concentrations of GPx mimics. j) Comparative GPx‐like activity.

Usually, under physiological condition, superoxide dismutase (SOD), as an essential antioxidant enzyme, catalyzes the dismutation of superoxide radical (O_2_·^‐^) into hydrogen peroxide (H_2_O_2_) and oxygen (O_2_), avoiding the further harm of secondary free radicals, in which accompanied by (ONOO‐) generation, and then converting into nontoxic H_2_O and O_2_ by catalase (CAT).^[^ ^36^, ^37^ ^]^ **Figure** **3**a shows the enzyme‐like properties of Fe_2_‐NCs. First, the SOD activity of the prepared nanozyme was evaluated by a WST‐8 assay system, which could react with O_2_·^‐^ rooting from xanthine and xanthine oxidase (XOD) reaction to form water‐soluble formazan dyes with an absorption wavelength of 450 nm (Figure 3b). Fe_2_‐NCs can obviously inhibit the rate of formazan production (inhibition rate: 94.30%) than Fe_1_‐NCs (50.50%) and NCs (0.032%), demonstrating the capacity of Fe_2_‐NCs to scavenge superoxide anions effectively (Figure S19, Supporting Information). As displayed in Figure 3c, the inhibition rate of formazan formation exhibited a dose‐dependent behavior. The IC_50_ value for Fe_2_‐NCs was determined to be 20.30 µg mL^−1^, significantly lower than that of Fe_1_‐NCs (>100 µg mL^−1^), highlighting the superior SOD‐like activity of Fe_2_‐NCs than Fe_1_‐NCs, which can further be expounded by Figure S20 (Supporting Information). Furthermore, Fe_2_‐NCs show a lowest response signal at 560 nm than the control group (Ctrl) and the Fe_1_‐NCs, suggesting that Fe_2_‐NCs own an enhanced SOD‐like activity and a greater capacity for scavenging O_2_·^‐^. As illustrated in Figure S21 (Supporting Information), Fe_2_‐NCs exhibit SOD mimicking activity (8645.2 U mg^−1^) that are comparable to that of natural SOD (Sigma, CAS:9054‐89‐1), while Fe_1_‐NCs and NCs show 5879.2 and 382.1 U mg^−1^, respectively. The aforementioned results indicate that Fe_2_‐NCs represent a promising candidate for SOD‐mimicking application with a progressive enhancement upon the incorporation of the Fe─Fe structure.

The obtained Fe_2_‐NCs also show the CAT performance, as shown in Figure S22 (Supporting Information). After uniformly mixing H_2_O_2_ with the prepared samples, the absorbance value of H_2_O_2_ at 240 nm was measured over time to quantitatively compare the decomposition ability of the prepared sample toward H_2_O_2_. After 120 min of reaction, the response absorbance of the reaction solution of the Fe_2_‐NCs, Fe_1_‐NCs, and NCs at 240 nm decreases from 18.05 to 1.98 and 8.15, indicating that Fe_2_‐NCs have a stronger kinetic rate to decompose H_2_O_2_ (Figure 3d). Figure 3e shows that H_2_O_2_ decomposition on Fe_2_‐NCs and Fe_1_‐NCs follows Michaelis‐Menten kinetics, and the K_m_ value of Fe_2_‐NCs for H_2_O_2_ is about half than that of Fe_1_‐NCs, and far below than that of natural CAT enzyme (CAS:9001‐05‐2, Aladdin), indicating that Fe_2_‐NCs has a more substantial adsorption effect on H_2_O_2_ (Table S4, Supporting Information). The V_max_ value of Fe_2_‐NCs is 1.74 times higher than that of Fe_1_‐NCs, and 2.64‐fold higher than that of natural CAT enzyme, implying that Fe_2_‐NCs exhibit a higher binding affinity for H_2_O_2_ and can convert H_2_O_2_ to O_2_ more efficiently. Figure S23 (Supporting Information) shows that the introduction of Fe_2_‐NCs into the H_2_O_2_ solution resulted in a noticeable increase in bubble formation, and the oxygen concentration in the solution exhibited a significant rise over time. Furthermore, the CAT‐like activity of Fe_2_‐NCs was calculated to be 85.31 U µg^−1^, which is 1.54 times higher than that of Fe_1_‐NCs (55.43 U µg^−1^, Figure S24, Supporting Information). The amount of O_2_ generation over Fe_2_‐NCs is assessed by using a dissolved oxygen analyzer, as exhibited in Figure 3f. Fe_2_‐NCs can produce ≈15.52 µmol of O_2_ from 100 mm H_2_O_2_ within 400 s, threefold higher than the 5.08 µmol produced by Fe_1_‐NCs. The CAT‐like activity of Fe_2_‐NCs was further measured by electron paramagnetic resonance experiments (Figure S25, Supporting Information).^[^ ^14^ ^]^ Compared with Fe_1_‐NCs, Fe_2_‐NCs can effectively suppress the formation of hydroxyl radicals (·OH) in the Fenton reaction, demonstrating that Fe_2_‐NCs possess a more heightened CAT‐like activity and a superior ability to scavenge H_2_O_2_.

The oxidase‐like (OXD) activity of the synthesized nanozymes was investigated as shown in Figure S26 (Supporting Information). After the reaction, the absorbance of ox‐TMB over Fe_2_‐NCs at 652 nm can reach 2.25, while Fe_1_‐NCs and NCs reach 1.58 and 0.13, respectively. After 60 min of reaction, the absorbance of the blue product generated by TMB catalyzed by Fe_2_‐NCs was 2.28, which is 1.82 times that of Fe_1_‐NCs (1.25, Figure 3g). The OXD activity value of Fe_2_‐NCs was calculated to be 93.9 U µg^−1^, 1.43 times than that of Fe_1_‐NCs (65.6 U µg^−1^, Figure S27, Supporting Information). Figure S28 (Supporting Information) shows the time dependent increase of the characteristic absorbance about oxidation of TMB, in which Fe_2_‐NCs exhibits a stronger catalytic ability than Fe_2_‐NCs. The OXD‐like activity of Fe_2_‐NCs follows the typical Michaelis‐Menten kinetics (Figure 3h), demonstrated by the corresponding comparison of the blue changes (Figure S29a,c, Supporting Information), catalytic activity (Figure S29b,d, Supporting Information). The K_m_ and V_max_ values further indicate that Fe_2_‐NCs possess a higher OXD activity than Fe_1_‐NCs (Table S5, Supporting Information). The OXD reaction rates over Fe_2_‐NCs are 4.07 µmol (L min^−1^)^−1^ (Figure S30, Supporting Information), which is 3.18 times higher than that of Fe_1_‐NCs (1.28 µmol (L min^−1^)^−1^). The above results demonstrate that Fe_2_‐NCs possess a more heightened OXD‐like activity and a superior ability to catalyze the oxidation of substrates into H_2_O or H_2_O_2_ than Fe_1_‐NCs.

The simulated catalytic activity of glutathione peroxidase (GSH‐Px) was also evaluated. As shown in Figure 3i, the GSH‐Px activity of Fe_2_‐NCs is 34.90 µm min^−1^, much higher than that of Fe_1_‐NCs (23.58 µm min^−1^) and NCs (7.49 µm min^−1^). The reaction rate shows that Fe_2_‐NCs is 90.38 mm min^−1^, 1.55 times higher than that of Fe_1_‐NCs (58.34 mm min^−1^, Figure 3j), revealing a higher GSH‐Px‐like activity and a superior ability to catalyze the oxidation of GSH into the nontoxic glutathione disulfide (GSSG) of Fe_2_‐NCs. Long‐term stability is another performance index for evaluating the nanozymes.^[^ ^38^ ^]^ As shown in Figure S31a–c (Supporting Information), the Fe_2_‐NCs can keep its initial activity in SOD‐, CAT‐, and GSH‐Px‐like activities test for 30 d, far more than natural enzymes. Meanwhile, the activity of Fe_2_‐NCs can up to the optimum performance within pH ranges from 5 to 9 (Figure S31d–f, Supporting Information). These results indicate that Fe_2_‐NCs exhibit natural‐enzyme‐comparable, SOD‐CAT‐GSH‐Px mimicking activity, and its stability is superior to that of the natural enzymes.

### 2.3 Mechanisms and Theoretical Simulations of Fe_2_‐NCs

**Figure 4:**
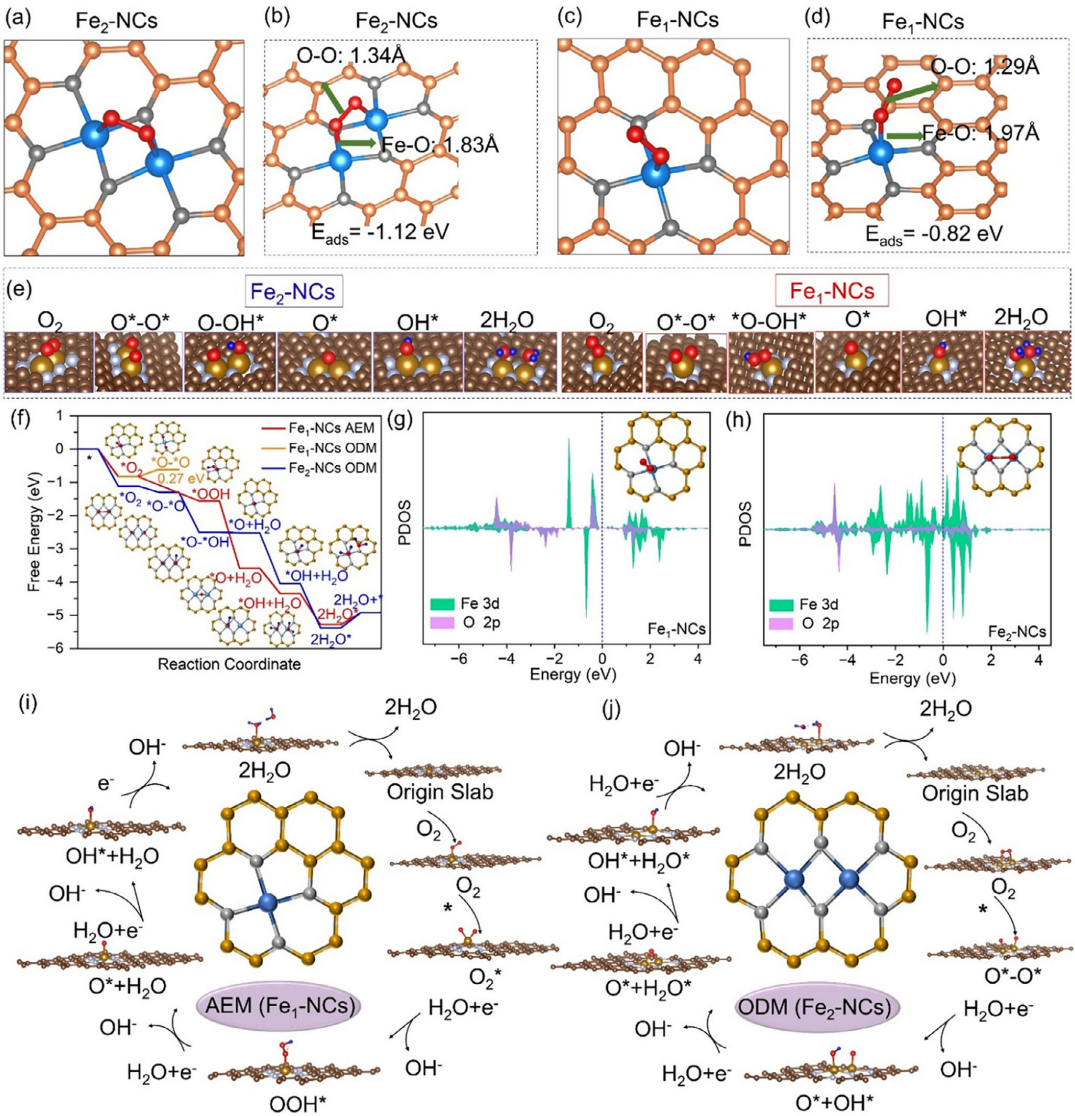
Atomic‐scale oxygen interaction mechanisms in Fe_1_/Fe_2_‐NCs catalytic systems. DFT‐optimized O_2_ adsorption configurations on Fe_2_‐NCs a,c) and Fe_1_‐NCs b,d): Topological electron density distributions (top view, a‐b); Charge transfer profiles (side view, c‐d). e) Adsorbed behavior of transition state (O_2_, O^*^–O^*^, O–OH^*^, O^*^, OH^*^ and H_2_O) in catalytic process. f) Comparative Gibbs free energy landscapes under dual catalytic pathways: AEM (red lines) vs ODM (blue lines).,g‐h) PDOS analysis for Fe‐3d and O‐2p orbital interactions: Fe_1_‐NCs (g) vs Fe_2_‐NCs (h). The Fermi level indicated by a dashed line at 0 eV. i,j) Schematic reaction coordinates: AEM‐mediated 4‐electron pathway (i) with sequential O‐O bond elongation; ODM‐driven 2‐electron route (j) involving direct O_2_ dissociation.

To further elucidate the relationship between the atomic‐scale configuration and the superior enzyme mimetic properties of the designed nanozymes, DFT simulations were conducted to compute the reaction free energy of H_2_O production over Fe_2_‐NCs and Fe_1_‐NCs. O_2_·^−^, acting as a Brønsted base, typically exists in biological fluids as the hydroperoxyl radical (HO_2_·).^[^ ^39^ ^]^ Theoretical calculations suggest that the adsorbed O_2_ on Fe_2_ sites of Fe_2_‐NCs can accelerate the cleavage of O─O bonds. The reason is that more electron can transfer into the vacant orbitals of O_2_ for better activation. **Figure** **4**a–d shows an adsorption model of O_2_ molecules on the synthesized nanozymes. The adsorption energies of O_2_ on Fe_2_‐NCs and Fe_1_‐NCs are −1.12 and −0.82 eV. In this case, the Fe─O bonds are 1.83 and 1.97 Å for Fe_2_‐NCs and Fe_1_‐NCs, respectively, indicating that the adsorption process at the atomic sites is most favorable on the Fe_2_ dimer.

The O─O bond of O_2_ adsorbed on Fe_1_‐NCs is ≈1.29 Å, attributed to superoxide, while O_2_ on Fe_2_‐NCs tends to form redox‐like structures with a significantly longer O─O bond of 1.34 Å, indicating that the redox‐like structures of Fe_2_‐NCs can elongate O─O bond distances, accelerating the cleavage of O─O bonds. Generally, the SOD‐like activity of nanozymes involves four fundamental steps. The transition state simulations (Figure 4e,f) disclose that the synergistic effect in the Fe_2_‐NCs will lower the reaction energy barrier for the intermediate release of O_2_ or H_2_O in comparison with Fe_1_‐NCs. The reduction in energy barrier, coupled with the enhanced reaction rates at every step, was identified as the primary factor to the improved reaction activity. Meanwhile, the PDOS results reveal a significant overlap between the 3d orbitals of Fe and the 2p orbitals of O at the Fe_2_ dimer, accounting for the strong activation capacity of O_2_ (Figure 4g,h).

Based on the above simulated results, the reaction pathway of O_2_ activation and conversion on Fe_2_‐NCs and Fe_1_‐NCs is illustrated as shown in Figure 4i,j. For Fe_2_‐NCs, the adsorbed O_2_ preferentially dissociates, rather than forming OOH^*^ as an intermediate. Consequently, the oxygen reduction reaction on Fe_2_‐NCs follows the oxygen dissociation mechanism (ODM), wherein the intermediates evolve sequentially from O^*^ to OH^*^ and finally to H_2_O^*^. This pathway is more favorable under mild reaction conditions, as it facilitates a smoother electron transfer process and is aligned with the molecular mechanistic requirements for efficient catalysis. On the contrary, the oxygen reduction pathway on Fe_1_‐NCs was simulated using the adsorption evolution mechanism (AEM), where the intermediate underwent structural rearrangement and relaxation upon O_2_ adsorption, eventually evolving into OOH^*^, O^*^ and OH^*^. Therefore, due to the low oxidation state of the Fe sites in Fe_2_‐NCs and strong adsorption capacity for O_2_, the reaction mechanism on Fe─Fe sites shifts from an AEM to ODM, accelerating the overall reaction rate, making the process more favorable for oxygen reduction and leading to enhanced SOD‐ and CAT‐like catalytic activities.

### 2.4 The In Vitro Ameliorates Oxidative Stress and Mitigates Mitochondrial Dysfunction Efficacy of Fe_2_‐NCs

**Figure 5:**
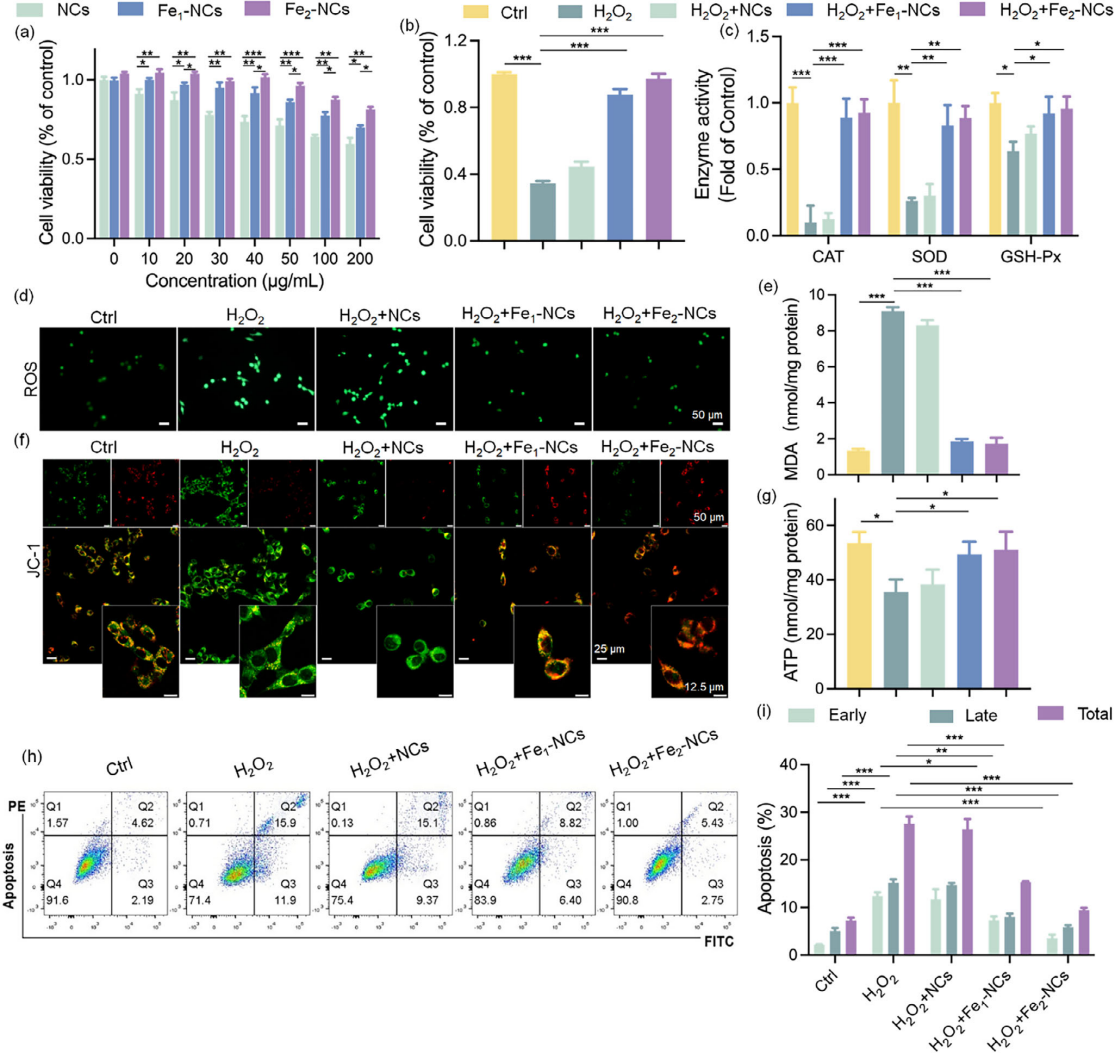
In vitro biocompatibility and antioxidative activities of the synthesized nanozyme on ATDC5 cells. a) Cytocompatibility assessment of ATDC‐5 cells exposed to escalating concentrations (0–200 µg mL^−1^) of Fe_2_‐NCs, Fe_1_‐NCs, and NCs via CCK‐8 assay (*n* = 6 biological replicates). b) Oxidative stress rescue efficacy: Cell viability under H_2_O_2_ (500 µm) challenge with/without nanozyme cotreatment (*n* = 6). c) Nanozyme‐mimetic catalytic kinetics quantification: CAT (H_2_O_2_ decomposition rate at 240 nm), SOD (WST‐8‐based O_2_^‐^· scavenging), GPx (NADPH oxidation at 340 nm) activities (*n* = 3). d) Intracellular ROS visualization via DCFH‐DA fluorescence under oxidative stress modulation (Scale bar: 50 µm, *n* = 3). e) MDA content of cells with different treatments. (*n* = 3). f) Mitochondrial polarization states analyzed via JC‐1 probe. g) Cellular bioenergetics profiling through ATP luminescence assay (*n* = 3). h,i) Annexin V‐FITC/PI dual‐staining apoptosis assay (*n* = 3). Data expressed as mean ± SD. ^*^ *p*<0.05, ^**^ *p*<0.01, ^***^ *p*<0.001 (One‐way ANOVA with Tukey's post hoc for multi‐group analysis).

To further validate the capacity of Fe_2_‐NCs in scavenging ROS and inhibiting apoptosis, in vitro cellular experiments were conducted. Following a 24‐h incubation of ATDC5 cells with escalating concentrations of the synthesized nanozymes, a noticeable reduction in cell viability was observed. The extent of cytotoxicity exhibited a concentration‐dependent relationship with the nanozyme treatment. The group co‐incubated with Fe_2_‐NCs exhibits higher cell survival rates, maintaining a cell viability of 81.5% at a high concentration of 200 µg mL^−1^, higher than thses of groups co‐incubated with NCs (59.6%) and Fe_1_‐NCs (69.9%), suggesting that Fe_2_‐NCs has a superior biocompatibility to cells (**Figure** **5**a). Hemolytic assessment is critical for nanomaterials with potential blood contact during administration or systemic distribution.^[^ ^40^, ^41^ ^]^ The hemolysis percentage quantifies erythrocyte membrane damage, where >5% indicates a significant risk of thrombosis and inflammation. The hemolytic properties of rat erythrocytes in Figure S32 (Supporting Information) show that Fe_2_‐NCs exhibit an absence of significant hemolysis. To assess cellular uptake characteristics in ATDC5 cells, RhB‐PEG@Fe_2_‐NCs was synthesized (Figure S33, Supporting Information). Laser confocal microscopy images reveal a dose‐dependent and time‐dependent increase in cellular uptake (Figures S34 and S35, Supporting Information). These findings collectively demonstrate that the prepared nanozymes exhibit excellent biocompatibility and are promising candidates for further application in biological systems. And then, H_2_O_2_ was utilized to establish an in vitro model of oxidative injury to explore the potential therapeutic applications over Fe_2_‐NCs in the context of oxidative stress‐related diseases, particularly in their ability to mitigate cellular damage and improve cell viability. Fe_2_‐NCs show the best ability in attenuating H_2_O_2_‐induced oxidative damage, significantly reducing intracellular ROS levels, and thereby enhancing cell viability (Figure 5b), which highlights efficacy as modulators of cellular resilience against oxidative damage.

Figure 5c shows the efficient restoration ability of SOD, CAT, and GSH‐Px activities in chondrocytes subjected to oxidative stress induced by H_2_O_2_ over the Fe_2_‐NCs, which can provide robust cytoprotective effects, mitigating H_2_O_2_‐induced oxidative damage, supporting the maintenance of cellular redox homeostasis, and ultimately enhancing cell viability and function. Figure 5d shows the intracellular ROS scavenging capacity of synthesized nanozymes, in which the Fe_2_‐NCs present a negligible fluorescence due to the low ROS levels compared with Fe_1_‐NCs, and NCs, indicating the superior ROS‐scavenging efficacy of Fe_2_‐NCs. The results document that the high‐content H_2_O_2_ will significantly elevate intracellular malondialdehyde (MDA) levels. The antioxidant properties of Fe_2_‐NCs are recorded in Figure 5e by substantially reducing MDA formation, and alleviating the cellular damage induced by oxidative stress.

As we know, excessive ROS not only contributes to inflammation but also induces chondrocyte apoptosis and mitochondrial dysfunction in OA, accelerating disease progression.^[^ ^42^, ^43^ ^]^ The protective effect of the synthesized nanozymes on mitochondrial health were monitored as shown in Figure 5f. In compared with Fe_1_‐NCs, and NCs, the Fe_2_‐NCs group exhibits trace green fluorescence and a pronounced red fluorescence signal, closely resembling the baseline, showing the reinforcement of its potent ability to safeguard mitochondrial function against H_2_O_2_‐induced cellular injury. To further evaluate the protective effects of Fe_2_‐NCs on mitochondrial function, we assessed mitochondrial membrane potential (ΔΨm) using JC‐1 staining. As shown in Figure 5f and Figure S36 (Supporting Information), H_2_O_2_ exposure markedly reduced ΔΨm, with a significant decrease in the green/red fluorescence ratio. Here, Fe_2_‐NCs treatment effectively reversed this depolarization, restoring the ratio to near‐control levels, significantly mitigating the H_2_O_2_‐induced ΔΨm loss.

To ensure reliable comparison of fluorescence intensity in cellular ROS and mitochondrial membrane potential assays, we further performed nuclear staining using Hoechst 33342, a dye with superior permeability in live cells, as DAPI is less suitable for live‐cell imaging. Hoechst 33342 staining enabled clear identification of nuclei, thereby confirming the consistency of cell numbers across different groups. Additionally, bright‐field images were captured in parallel to further validate the comparability of fluorescence signals. The representative images are provided in Figures S37 and S38 (Supporting Information). These complementary data strengthen the robustness of the fluorescence analyses and confirm that the observed reduction of ROS and restoration of mitochondrial membrane potential following Fe_2_‐NCs treatment are not confounded by variations in cell number. To further validate these findings, we performed JC‐1–based flow cytometry to quantitatively assess ΔΨm (Figures S39 and S40, Supporting Information). The results were consistent with immunofluorescence observations, further confirming that Fe_2_‐NCs preserve mitochondrial polarization under oxidative stress. Notably, this recovery of ΔΨm coincided with elevated ATP levels and upregulated COXIV expression, indicating that Fe_2_‐NCs protect not only mitochondrial structure but also bioenergetic capacity.

The higher H_2_O_2_ content will restrict cellular bioenergetics and impair mitochondrial function through decreasing the adenosine 5′‐triphosphate (ATP) concentration.^[^ ^44^ ^]^ Figure 5g shows that Fe_2_‐NCs can led to a notable recovery of ATP levels, underscoring its enhanced protective potential. To assess the impact of oxidative stress on chondrocyte apoptosis, flow cytometry was utilized to analyze H_2_O_2_‐induced apoptotic events in ATDC5 cells (Figure 5h,i). The Fe_2_‐NCs exhibit significantly reduced apoptosis rates, ≈10.48%, far blew than that of Fe_1_‐NCs (15.69%) and NCs (22.45%), indicating that Fe_2_‐NCs effectively protect chondrocytes from oxidative stress‐induced apoptosis, thereby significantly decreasing the incidence of cell death. Fe_2_‐NCs induce minimal cell death in the cells containing H_2_O_2_ (Figure S41, Supporting Information), exhibiting no apparent toxicity and maintaining normal cell morphology in comparison to the control group. With the increasing of the Fe_2_‐NCs dosage, the cell density progressively increases (Figure S42, Supporting Information), disclosing that the synthesized Fe_2_‐NCs effectively neutralize excess ROS, thereby alleviating oxidative stress and substantially reducing both apoptosis and cell death. Beyond their strong ROS‐scavenging capacity, Fe_2_‐NCs exhibit selective accumulation in ROS‐activated cells within the osteoarthritic microenvironment. This selectivity arises from both material design and cellular mechanisms. From the structural perspective, the Fe─Fe diatomic sites provide optimized electronic configurations that enhance catalytic performance. DFT calculations revealed stronger O_2_ adsorption (−1.12 vs −0.82 eV for Fe_1_‐NCs) and elongation of the O─O bond (1.34 Å), favoring activation and cleavage. The synergistic Fe─Fe sites shift the oxygen reduction pathway from the adsorption–evolution mechanism to the more efficient oxygen dissociation mechanism, thereby lowering the reaction barrier and accelerating electron transfer. These features explain the superior SOD‐ and CAT‐like activities of Fe_2_‐NCs. At the cellular level, Fe_2_‐NCs target ROS‐activated cells through electrostatic interactions and stress‐enhanced uptake. Under oxidative stress, externalization of phosphatidylserine imparts a strong negative surface charge, to which positively charged Fe_2_‐NCs (+26.81 mV) preferentially bind.^[^ ^45^, ^46^, ^47^ ^]^ Concurrently, ROS stress increases membrane permeability and endocytic activity, further promoting nanomaterial uptake.^[^ ^48^, ^49^ ^]^ Consistently, H_2_O_2_‐stimulated chondrocytes exhibited markedly higher Fe_2_‐NCs internalization compared with normal cells (Figure S43, Supporting Information). Together, these results highlight that Fe_2_‐NCs not only scavenge ROS efficiently but also selectively enrich in ROS‐overexpressing cells via complementary physicochemical and biological mechanisms. This dual targeting strategy ensures precise intervention within the inflammatory microenvironment of osteoarthritis, thereby enhancing therapeutic efficacy. These results underscore that Fe_2_‐NCs not only significantly mitigate oxidative stress‐induced cellular damage, but also promote the restoration of mitochondrial function by enhancing cellular energy metabolism. Consequently, Fe_2_‐NCs play a crucial role in protecting chondrocytes from apoptosis. Further analysis reveals that Fe_2_‐NCs exhibit a more pronounced effect in preserving mitochondrial integrity and maintaining cellular energy homeostasis, highlighting their considerable potential as a therapeutic agent in restoring mitochondrial function under oxidative stress conditions.

### 2.5 Molecule Mechanisms Underlying the Therapeutic Effects of Fe_2_‐NCs in OA

**Figure 6:**
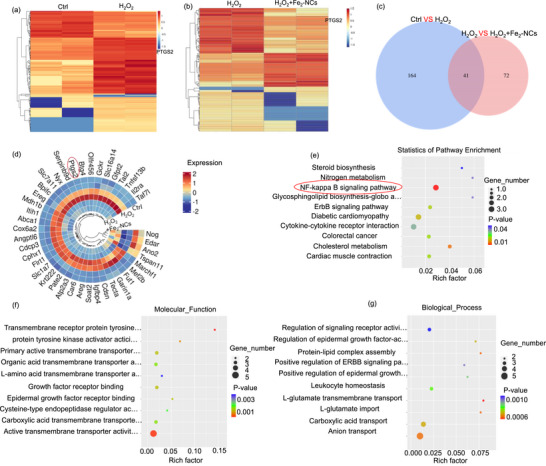
RNA sequencing analysis of ATDC5 cells with H_2_O_2_ induction and Fe‐atom nanozyme treatment. a) Heat maps of DEGs in Ctrl vs H_2_O_2_ group and H_2_O_2_ vs H_2_O_2_ + Fe_2_‐NCs group, where red indicates upregulation and blue indicates downregulation of protein‐coding genes. b) Venn diagram of the intersection of DEGs in Ctrl vs H_2_O_2_ group and H_2_O_2_ vs H_2_O_2_ + Fe_2_‐NCs group. c) Circular Heatmap of DEGs obtained by the intersection of two groups. d) KEGG enrichment analysis of intersection DEGs. e–g) GO enrichment analysis of intersection DEGs from biological processes and molecular functions.

To gain deeper insights into the molecular mechanisms underlying the therapeutic effects of Fe_2_‐NCs in OA, RNA transcriptome profiling was performed on three experimental groups: normal chondrocytes (NCs group), H_2_O_2_‐induced chondrocytes (H_2_O_2_ group), and H_2_O_2_‐induced chondrocytes treated with Fe_2_‐NCs (H_2_O_2_+Fe_2_‐NCs group). Mapping clean readings to the reference genome, which are used to analyze gene expression levels in protein coding genes. **Figure** **6**a presents a clustering analysis of differentially expressed genes (DEGs). Statistical analysis of the DEGs reveals significant differences between the H_2_O_2_ and NC groups, with 205 DEGs identified (*p* <0.05), in which 154 are upregulated and 51 downregulated, suggesting that H_2_O_2_ will damage and degenerate of chondrocytes by modulating the expression of a range of genes. The H_2_O_2_ + Fe_2_‐NCs group only exhibits 113 DEGs (*p* <0.05) compared to the H_2_O_2_ group with 36 genes upregulate and 77 downregulate, indicating that Fe_2_‐NCs intervention notably reshapes the gene expression profile altered by oxidative stress, potentially alleviating chondrocyte pathological changes by inhibiting or repairing molecular pathways associated with cellular damage.

Further cross‐analysis of DEGs reveals 41 intersecting DEGs, serving as potential therapeutic targets of Fe_2_‐NCs in OA treatment (Figure 6b,c). A specific gene, cyclooxygenase‐2 (COX‐2, also known as PTGS2), is highlighted to exhibit a marked upregulation in the H_2_O_2_ group and a significant downregulation in the H_2_O_2_+Fe_2_‐NCs group, revealing that Fe_2_‐NCs exerts their anti‐inflammatory effects through the inhibition of COX‐2 expression, potentially mitigating joint degeneration. Pathway analysis of DEGs on H_2_O_2_ + Fe_2_‐NCs group was subsequently performed using the Kyoto Encyclopedia of Genes and Genomes (KEGG) database (Figure 6d), with enrichment scores plotted along the horizontal axis. The enrichment analysis results reveal that the NF‐κB signaling pathway exhibits the highest enrichment score. Combining the critical involvement of NF‐κB activation in the progression of cartilage damage and the pathogenesis of arthritis, a conclusion can be obtained that Fe_2_‐NCs can exert their anti‐inflammatory and chondroprotective effects in OA by modulating the NF‐κB signaling pathway. The Gene Ontology (GO) analysis and cellular functions analysis (Figure 6e–g; Figure S44, Supporting Information) demonstrate that Fe_2_‐NCs will exert therapeutic effects in OA through multiple mechanisms, including the modulation of inflammatory signaling pathways, alleviation of oxidative stress, and restoration of cellular homeostasis.

**Figure 7:**
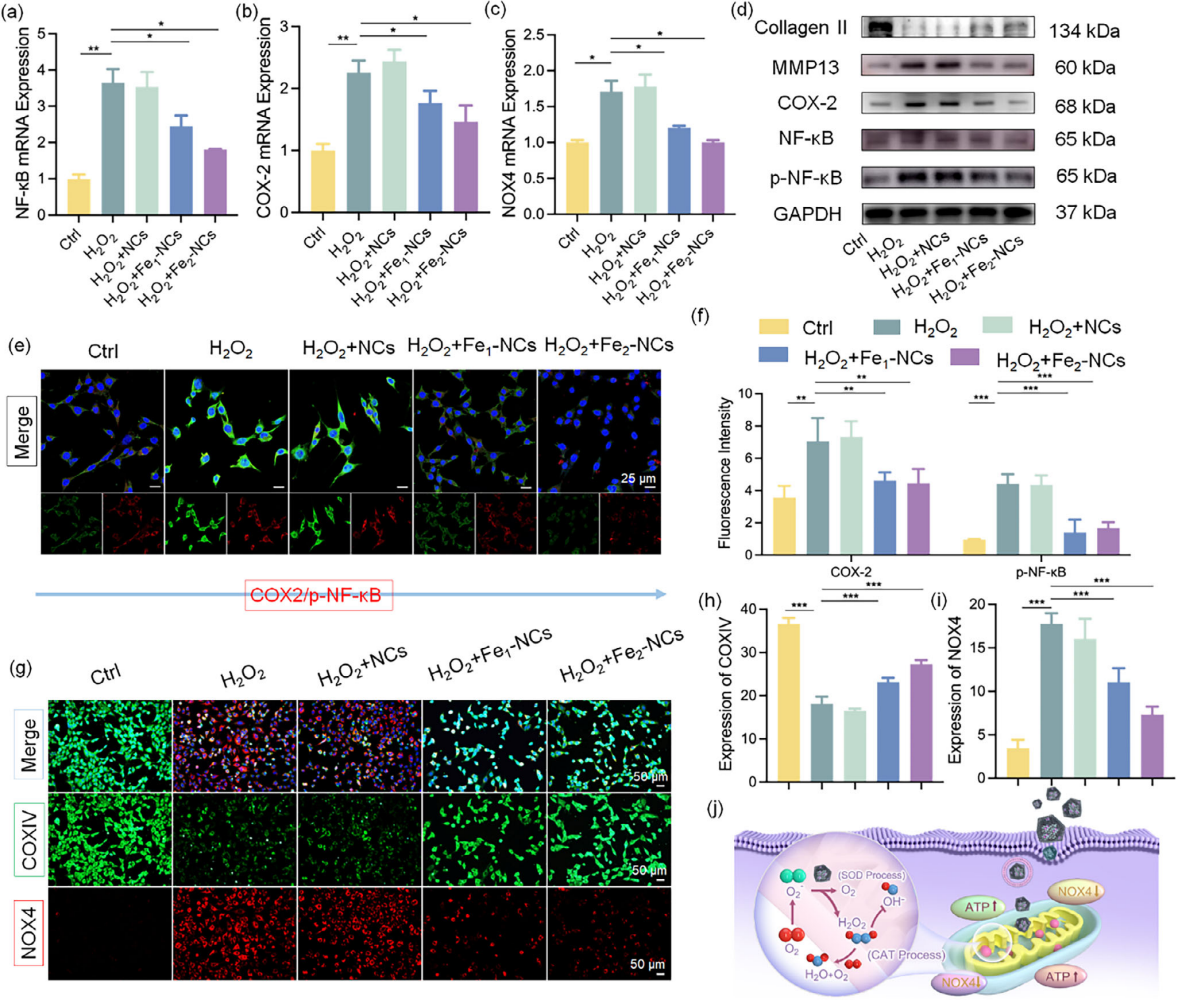
The synthesized nanozyme can effectively inhibit inflammation in chondrocytes through COX‐2 and mitigate mitochondrial damage in chondrocytes caused by oxidative stress via NOX4. a–c) qRT‐PCR quantification of inflammatory mediators (NF‐κB p65, COX‐2) and oxidative stress regulator (NOX4/NADPH oxidase 4) mRNA expression. Data normalized to β‐actin (*n* = 3 biological replicates). d) Western blot densitometric analysis of cartilage homeostasis markers: anabolic collagen II (COL2A1), catabolic MMP13, inflammatory COX‐2, and oxidative NOX4. GAPDH served as a loading control (*n* = 3). e,f) Confocal immunofluorescence imaging of phosphorylated NF‐κB p65 and COX‐2 subcellular localization with DAPI nuclear counterstain. Fluorescence intensity quantified via ZEN 3.0 software (Scale bar: 25 µm, *n* = 3). g–i) Super‐resolution Airyscan imaging of mitochondrial NOX4 and COXIV colocalization patterns. Pearson's coefficient calculated using ImageJ JACoP plugin (Scale bar: 25 µm, *n* = 3). j) Schematic of Fe_2_‐NCs’ catalytic cascade: From SOD‐mimetic O_2_^‐^· dismutation to H_2_O_2_ elimination via CAT‐like activity, and then NF‐κB/COX‐2 axis suppression. Statistical annotations: ^*^ *p*<0.05, ^**^ *p*<0.01, ^***^ *p*<0.001 (One‐way ANOVA with Šidák correction for multi‐group analysis).

The OA pathogenesis is closely linked to inflammatory responses, with the upregulation of inflammatory mediators serving as key indicators in the progression of the disease.^[^ ^50^ ^]^ The transcriptomic analyses further substantiate these observations to investigate the specific mechanisms involved in the inflammatory response. **Figure** **7**a,b shows the expression levels of phosphorylated nuclear factor kappa B (p‐NF‐κB) and COX‐2 quantified by polymerase chain reaction (qPCR). Notably, the qPCR results are in strong agreement with the transcriptomic findings, emphasizing the crucial role of the synthesized nanozymes in significantly reducing COX‐2 expression and inhibiting NF‐κB pathway activation. To further elucidate the role of ROS in OA, the expression of NADPH oxidase 4 (NOX4) was specifically investigated. qPCR analysis results demonstrate that Fe_2_‐NCs effectively attenuate NOX4 expression, reducing ROS generation and mitigating mitochondrial damage (Figure 7c). Western blot analyses and immunofluorescence provide further validation of the above results (Figure 7d–f). The booming expression of both p‐NF‐κB and COX‐2 induced by H_2_O_2_ can effectively reverse by Fe_2_‐NCs nanozyme. By mitigating ROS accumulation and suppressing the activation of inflammatory signaling pathways, the synthesized Fe_2_‐NCs nanozyme demonstrates a promising therapeutic potential in combating inflammation and protecting cartilage from degeneration.

### 2.6 Suppression of the COX‐2/NF‐κB Signaling Pathway and Mitigation of Mitochondrial Dysfunction Induced by NOX4 Activation of Fe_2_‐NCs

During the initiation and progression of OA, the over‐degradation of type II collagen (COL2) serving as a defining marker of cartilage degeneration by matrix metalloproteinase‐13 (MMP‐13), will impair ECM remodeling and regeneration, further exacerbating cartilage degradation and accelerating the OA progression.^[^ ^51^, ^52^, ^53^ ^]^ As demonstrated in Figure 7d and Figure S45 (Supporting Information), H_2_O_2_ stimulation in chondrocytes significantly elevated MMP‐13 expression relative to the control group, thereby promoting COL2 degradation. Fe_2_‐NCs, a novel class of antioxidants, can effectively inhibit the H_2_O_2_‐induced upregulation of MMP‐13 in chondrocytes, thereby mitigating COL2 degradation and decelerating OA progression. Consequently, the expression levels of MMP‐13 and other inflammation‐associated proteins are significantly reduced, offering a promising therapeutic avenue for OA intervention. To verify that the observed results in p‐NF‐κB reflect true pathway activation rather than variations in total protein expression, the total NF‐κB levels in each group were further assessed (Figure S46, Supporting Information). The results showed that total NF‐κB expression remained relatively constant across all treatment conditions, confirming that the differences in p‐NF‐κB levels were due to phosphorylation changes rather than altered protein abundance.

Usually, an abnormal increase in ROS levels leads to mitochondrial dysfunction, which subsequently triggers chondrocyte apoptosis, amplifies inflammatory cascades, and accelerates the ECM degradation of the cartilage.^[^ ^54^, ^55^ ^]^ The mitochondrial integrity was investigated by using cytochrome c oxidase subunit IV (COXIV) as a mitochondrial marker (Figure 7g–i). In chondrocytes subjected to H_2_O_2_‐induced oxidative stress, a marked upregulation of NOX4 expression is observed, concomitant with a significant reduction in COXIV levels, indicating mitochondrial damage in response to oxidative stress. Interestingly, a substantial reduction in NOX4 expression and a notable increase in COXIV levels are observed in the Fe_2_‐NCs group, implying that Fe_2_‐NCs provide effective protection against mitochondrial damage induced by ROS, suggesting that its mitochondrial protective effects are attributed to a comprehensive restoration of mitochondrial function. It is concluded that Fe_2_‐NCs nanozyme under H_2_O_2_ environment can effectively exert SOD‐like and CAT‐like effects to reduce oxidative stress‐induced mitochondrial damages, protect mitochondrial functions, and avoid energy deficiency via normalizing ATP and downregulating NOX4, as illustrated in Figure 7j.

### 2.7 In Vivo Therapeutic Effects of Fe_2_‐NCs in OA

**Figure 8:**
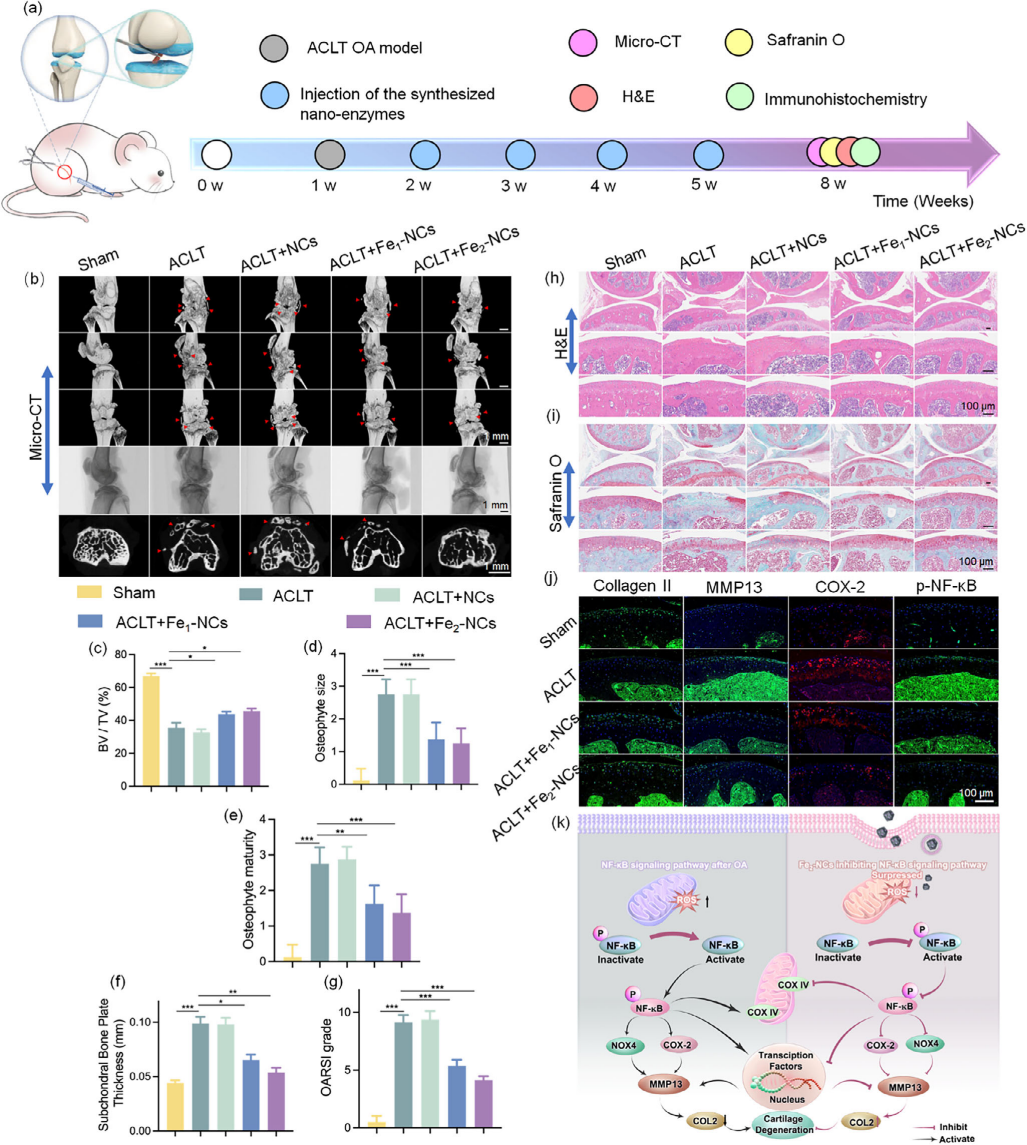
In vivo therapeutic evaluation of Fe_2_‐NCs in ACLT‐induced OA. a) Experimental workflow encompassing ACLT‐OA modeling, nanozyme intervention protocols, and multimodal therapeutic outcome assessment. b) Multiscale micro‐CT visualization: 3D reconstructed joint morphology (top), sagittal plane tomography (middle), and transverse section architecture (bottom). The red arrow was pointing to the osteophyte. Scale bar: 1 mm (*n* = 6 biological replicates per cohort). c–g) Quantitative microarchitectural parameters: Osteoarthritis Research Society International (OARSI) histopathology scores; subchondral trabecular morphometry (BV/TV ratio, plate thickness); osteophyte quantification (volume, maturity grade). Data represent mean ± error (*n* = 6). h,i) Histomorphological characterization: H&E staining (cartilage integrity assessment); Safranin O/Fast Green staining (proteoglycan distribution). Scale bar: 100 µm (*n* = 6). j) Immunohistochemical profiling of cartilage extracellular matrix biomarkers: Collagen II (anabolism), MMP13 (catabolism), COX‐2 (inflammation), phospho‐NF‐κB p65 (signaling activation). Scale bar: 100 µm (*n* = 6). ^*^ *p*<0.05, ^**^ *p*<0.01, ^***^ *p*<0.001 (One‐way ANOVA with Tukey's post hoc for multi‐group analysis). k) Mechanistic schema: Fe_2_‐NCs‐mediated joint protection through ROS scavenging and NF‐κB signaling suppression in ACLT‐induced OA pathogenesis.

To further explore the therapeutic potential of the synthesized nanozymes on OA, an OA mouse model was established via anterior cruciate ligament transection (ACLT, **Figure** **8**a). Following successful induction of the model, five experimental groups were formed Sham, ACLT, ACLT + NCs, ACLT + Fe_1_‐NCs, and ACLT + Fe_2_‐NCs. In this study, all samples were collected at 8 weeks post‐surgery, and a 3D model of the murine knee joint was generated using Micro‐CT scanning and reconstruction techniques, which facilitates a thorough and visually informative assessment of the knee joint's conditions (Figure 8b). Osteophytes are usually associated with joint degeneration in OA. Micro‐CT analysis shows obvious osteophyte formation at the joint edge of OA (red arrow), which is a sign of structural degeneration. But after treatment with Fe_2_‐NCs, its content significantly decreased. Besides that, cartilage degeneration was quantitatively evaluated using the OARSI scoring system.^[^ ^56^, ^57^ ^]^ Following these standardized methods, we systematically quantified synovial thickness and inflammatory cell infiltration, as well as tibial cartilage remodeling parameters, including trabecular number and area. The quantitative analysis of the Micro‐CT images revealed that ACLT surgery resulted in significant alterations in key osteological features. In specific, the Fe_2_‐NCs group (bone volume fraction: 45.63, osteophyte size: 1.25, osteophyte maturity: 1.38, subchondral bone plate thickness: 0.0539) exhibits significant therapeutic improvements in bone volume fraction and osteophyte size, as well as a notable reduction in cartilage degradation (Figure 8c–f), indicating that the Fe_2_‐NCs markedly improved subchondral bone microarchitecture. Besides that, our results demonstrate that Fe_2_‐NCs also effectively attenuate synovitis, thereby reinforcing their potential for treating osteoarthritis as a whole‐joint disease. Quantitative analysis of synovial tissue showed a significant reduction in inflammatory scores in the ACLT + Fe_2_‐NCs group compared with both ACLT and ACLT + Fe_1_‐NCs groups, underscoring the superior anti‐inflammatory efficacy of diatomic Fe_2_‐NCs (Figure S47, Supporting Information). Mechanistically, the robust ROS‐scavenging capacity of Fe_2_‐NCs alleviates oxidative stress in the synovial microenvironment, a critical driver of synovial hyperplasia and immune cell infiltration. Furthermore, by suppressing NF‐κB activation, Fe_2_‐NCs reduce the release of pro‐inflammatory cytokines, thereby disrupting the vicious cycle of oxidative stress and inflammation. Collectively, these findings indicate that Fe_2_‐NCs not only preserve cartilage matrix integrity but also directly mitigate synovial pathology, highlighting their multifaceted therapeutic potential in osteoarthritis management. In contrast, both the Fe_1_‐NCs (43.80, 1.38, 1.63, 0.0635) and NCs (32.78, 2.88, 2.75, 0.096) groups show a negligible reduction in knee OA symptoms. These results collectively underscore the beneficial therapeutic effects of the synthesized Fe_2_‐NCs nanozyme in the treatment of ACLT‐induced OA. Figure 8g shows the Osteoarthritis Research Society International (OARSI) scores of Fe_2_‐NCs + ACLT is ≈4.13, lower than that of other control groups, indicating the synthesized Fe_2_‐NCs nanozyme effectively mitigates cartilage damage and promotes recovery following ACLT‐induced injury.

The protective effects of the Fe_2_‐NCs nanozymes for cartilage were assessed by comprehensive histological analysis after 8 weeks post‐ACLT surgery (Figure 8h,i). Histological staining with H&E and Safranin O/Fast Green revealed substantial cartilage degradation in the knee joints of mice subjected to ACLT, exhibiting the characteristic features of OA. Specifically, irregular surface wear and erosive fissures are evident, accompanied by significant disruption of the cartilage matrix and cellular structure, further indicating profound impairment of the cartilage's natural repair mechanisms.^[^ ^58^ ^]^ These changes culminate in a marked thinning of the cartilage layer, a significant decrease in chondrocyte density, and a notable narrowing of the joint space. While Fe_2_‐NCs group demonstrates a significant reduction in ACLT‐induced cartilage damage. The overall cartilage layer in these groups was visibly thicker, with a notable restoration of the structural integrity of the cartilage tissue. The cellular arrangement appears more organized, and the chondrocyte count is significantly higher than in the ACLT group. Moreover, the joint space is substantially wider, supporting the conclusion that the synthesized nanozymes effectively mitigate cartilage damage and provide protective benefits to the joint structure.

To further verify the therapeutic differences between Fe_1_‐NCs and Fe_2_‐NCs in ACLT‐induced OA, we quantitatively analyzed the immunohistochemical staining results of Collagen II, MMP13, and p‐NF‐κB (Figure 8j; Figure S48, Supporting Information). The fluorescence intensity was specifically measured in the cartilage region to avoid potential interference from the subchondral bone. The quantitative results reveal that treatment with the synthesized nanozymes significantly reduced the expression of key markers associated with OA pathogenesis and cartilage degradation, including COX‐2, p‐NF‐κB, and MMP13, compared to the knee joints of mice that underwent ACLT surgery alone (Figure S49, Supporting Information). The expression of type II collagen was markedly upregulated in the knee joints of treated mice. These results suggest that the synthesized nanozymes alleviate ACLT‐induced OA by promoting cartilage matrix preservation, attenuating matrix degradation, and inhibiting inflammatory signaling. NF‐κB has been reported to be activated under the state of oxidative stress, activating the downstream pro‐inflammatory protein kinase COX‐2 and oxidative stress‐related protein NOX4 by phosphorylation.^[^ ^59^, ^60^ ^]^ Subsequently, p‐NF‐κB triggered inflammatory in a transcriptional and non‐transcriptional dependent manner (Figure 8k). Thus, it is plausibly speculated that Fe_2_‐NCs alleviates oxidative‐stress‐induced cellular mitochondrial damage and achieve OA repair by interfering with the NF‐κB signaling pathway.

Given the importance of in vivo biodistribution, we conducted comprehensive joint‐specific analyses to evaluate the intra‐articular retention of Fe_2_‐NCs (Figures S50 and S51, Supporting Information). ICP‐MS measurements of dissected joint compartments and major organs following intra‐articular injection revealed strong local retention, with ≈78% of the injected dose (0.78 µg Fe g^−1^ joint tissue) remaining at day 1. Notably, even at day 9, ≈29% of the injected dose (0.29 µg Fe g^−1^) was still detectable, indicating sustained intra‐articular persistence. In contrast, the systemic distribution was negligible, with the Fe accumulation in the liver and spleen both remaining at a relatively low level of injection dose, so as not to cause significant toxicity, granuloma formation, fibrosis, or other chronic inflammatory reactions, confirming minimal systemic exposure (Figure S55, Supporting Information). These findings demonstrate that Fe_2_‐NCs possess prolonged intra‐articular residence, thereby ensuring sustained local therapeutic activity. The combination of high joint retention and low off‐target distribution underscores their potential to deliver persistent anti‐inflammatory and antioxidant effects in situ, which is particularly advantageous for OA therapy.

To reconcile the apparent discrepancy between the in vitro and in vivo findings, we conducted additional mechanistic investigations. Gradient H_2_O_2_ assays (50–1000 µm) demonstrated that Fe_2_‐NCs exhibited significantly stronger ROS‐scavenging activity than Fe_1_‐NCs at physiologically relevant concentrations (<500 µm) (Figure S52, Supporting Information). However, under acute, high‐dose oxidative stress (≥500 µm H_2_O_2_), the difference between the two nanozymes became less discernible, likely due to a saturation effect whereby ROS clearance approaches a plateau independent of catalytic efficiency. Time‐resolved analyses further revealed that Fe_2_‐NCs not only achieved faster ROS elimination but also maintained prolonged scavenging capacity, consistent with their superior SOD‐like (IC_50_ = 20.30 vs >100 µg mL^−1^ for Fe_1_‐NCs) and enhanced CAT‐like activity (1.74‐fold) (Figure S53, Supporting Information). Moreover, ROS‐specific probe assays confirmed that Fe_2_‐NCs more effectively eliminated both O_2_•− and •OH, thereby providing direct evidence of their broad‐spectrum, multi‐enzyme–mimetic activity (Figures S54 and S55a–c, Supporting Information). Beyond catalytic performance, DFT analysis indicated that Fe_2_‐NCs preferentially follow a lower‐energy dual ^*^OOH pathway (−1.52 vs −2.54 eV for Fe_1_‐NCs), while their larger size and positive surface potential enhanced colloidal stability, dispersibility, and intra‐articular retention. These physicochemical advantages translated into pronounced in vivo efficacy: Fe_2_‐NCs sustained ROS clearance, protected against oxidative injury, and regulated downstream redox‐sensitive signaling pathways, including suppression of NF‐κB and MMP‐13. Importantly, Fe_2_‐NCs maintained superior antioxidant efficacy even at low doses (2 µg mL^−1^), as evidenced by reduced MDA levels and elevated SOD activity (Figure S56, Supporting Information).

Collectively, these findings suggest that the intrinsic advantages of Fe_2_‐NCs may not be fully captured under oversaturated in vitro but become evident in vivo, where oxidative stress is chronic, spatially heterogeneous, and intricately coupled with inflammatory, immune, and matrix‐related processes. Notably, the limitations of simplified in vitro oxidative models compared with complex physiological microenvironments have also been emphasized in recent nanozyme studies.^[^ ^39^, ^61^, ^62^, ^63^, ^64^, ^65^ ^]^ Taken together, our results demonstrate that the superior therapeutic efficacy of Fe_2_‐NCs arises from their optimized electronic structure and physicochemical stability, coupled with their ability to integrate catalytic ROS clearance with modulation of redox and inflammatory signaling under biologically relevant conditions.

### 2.8 In Vivo Biosafety and Toxicity of Fe_2_‐NCs

Biosafety and toxicity are critical considerations for the potential clinical translation of novel nanomaterials.^[^ ^66^ ^]^ Accordingly, the biological safety of the synthesized nanozyme was rigorously assessed in vivo. 8 weeks post‐ACLT surgery, a comprehensive analysis and histopathological evaluations of the blood and organs across all groups were conducted. Peripheral blood samples were collected following repeated intra‐articular injections and subjected to complete blood count analysis. The measured parameters included total and differential leukocyte counts (WBC, Neu#, Lym#, Mon#, Eos#, Bas#, Eos%, Bas%), erythrocyte indices (RBC, HCT, MCV, MCH, RDW‐CV, RDW‐SD), hemoglobin levels (HGB, MCHC), and platelet‐related indicators (PLT, MPV, PCT, PDW). All values in the Fe_2_‐NCs‐treated group remained within ±5% of the control group mean, with no statistically significant differences (ns, *p* >0.05) (Figure S57a–c, Supporting Information). These results indicate that Fe_2_‐NCs do not induce abnormalities in leukocyte, erythrocyte, or platelet counts, proportions, or morphology, nor do they affect hemoglobin concentration, thereby confirming their good biocompatibility and low systemic hematological toxicity in vivo.

It is noteworthy that a fundamental advantage of iron‐based nanomaterials lies in the fact that their ultimate degradation products are endogenous iron species—strictly regulated by the body's iron metabolism—rather than non‐degradable exogenous substances.^[^ ^67^, ^68^, ^69^, ^70^, ^71^, ^72^, ^73^, ^74^ ^]^ DLS analysis of Fe_2_‐NCs post‐degradation in 2 months revealed a primary particle size distribution in the range of 6–20 nm (Figure S58, Supporting Information), which suggests a predominant hepatic clearance mechanism in vivo. Specifically, circulating nanoparticles are phagocytosed by Kupffer cells in the liver, followed by biliary excretion of degradation products into the intestine, and eventual elimination through the fecal route.^[^ ^75^, ^76^, ^77^, ^78^, ^79^ ^]^ To further exclude any potential organ toxicity induced by the nanozyme during an 8‐week long‐term toxicity assessment, histological analysis of key organs (heart, liver, spleen, lungs, and kidneys) performed using H&E staining showed no signs of progressive toxicity, granuloma formation, fibrosis, or other chronic inflammatory responses (Figure S59, Supporting Information). These findings collectively support a controllable degradation and clearance pathway of Fe_2_‐NCs that is closely aligned with the body's physiological iron metabolism. Compared to many other non‐degradable nanomaterials, this mechanism significantly reduces the risk of long‐term accumulation, underscoring the superior biocompatibility and safety profile of Fe_2_‐NCs for chronic biomedical applications, and laying a solid foundation for subsequent clinical investigations.

## 3 Conclusion

In summary, this study demonstrates that homonuclear Fe─Fe dimer sites anchored on nitrogen‐doped porous carbon (Fe_2_‐NCs) provide a potent nanozymatic platform for mitigating oxidative stress and slowing OA progression. The Fe_2_‐NCs efficiently scavenge excess ROS through a co‐adsorption mechanism, mimicking key antioxidant enzyme activities. DFT reveals that their enhanced activity originates from the synergy between Fe─Fe dimer sites, which optimizes oxygen adsorption and O─O bond cleavage. In vitro and in vivo models, Fe_2_‐NCs protect chondrocytes from oxidative stress‐induced apoptosis, improve mitochondrial function by inhibiting NOX4 expression and restoring ATP production, and dampen the inflammatory response by downregulating COX‐2. Furthermore, this approach inhibits MMP‐13 upregulation and prevents Type II collagen degradation, thereby preserving joint homeostasis. Overall, these findings highlight the potential of Fe_2_‐NCs as a promising therapeutic strategy for OA by strengthening cellular defenses and inhibiting catabolic activity.

## 4 Experimental Section

### Synthesis of ZIF‐8

A precursor solution was prepared by dispersing Zn (NO_3_)_2_ 6H_2_O (1.19 g, 4 mmol) in a ternary solvent system comprising MeOH (12 mL), N,N‐dimethylformamide （DMF） (36 mL), and ethylene glycol (EG, 12 mL) through ultrasonication (40 kHz, 150 W) for 15 min (Solution A). Concurrently, 2‐mIM (1.314 g, 16 mmol) was dissolved in a binary solvent mixture of MeOH (8 mL) and DMF (12 mL) under magnetic agitation until complete dissolution (Solution B). The synthesis proceeded by dropwise addition of Solution A into Solution B under vigorous mechanical stirring (800 rpm) at ambient temperature (25±2 °C). The reaction mixture was maintained under continuous agitation for 24 h to facilitate coordination self‐assembly. The resultant colloidal product was isolated via high‐speed centrifugation (10000 × g, 5 min) and subjected to three sequential washing cycles with anhydrous methanol to remove unreacted precursors. Final purification was achieved through vacuum drying (70 °C, 0.1 MPa) for 12 h to obtain crystalline ZIF‐8 derivatives.

### Synthesis of NCs

The as‐synthesized ZIF‐8 precursor was loaded into a quartz crucible positioned at the constant temperature zone of a horizontal tubular furnace (GSL‐1700X, Hefei Kejing). A controlled pyrolysis protocol was implemented under inert atmosphere: The temperature was elevated to 950 °C at 5 °C min^−1^ ramp rate under continuous argon flow (99.99% purity, 100 sccm), maintained isothermally for 3 h, followed by passive cooling to ambient temperature. The resultant carbonized material was collected without post‐processing for subsequent characterization.

### Synthesis of Fe_2_(CO)_9_@ZIF‐8/Fe(acac)_2_@ZIF‐8

A homogeneous metal precursor solution (Solution A) was formulated by dissolving Zn(NO_3_)_2_ 6H_2_O (1.19 g, 4 mmol) and Fe_2_(CO)_9_ (11 mg, 0.03 mmol) in a ternary solvent system comprising DMF (36 mL), MeOH (12 mL), and EG (12 mL). Ultrasonication (40 kHz, 150 W) was applied at ambient temperature (25 °C) to ensure complete dissolution. Simultaneously, Solution B was prepared by dissolving 2‐mIM (1.314 g, 16 mmol) in a binary solvent mixture of DMF (12 mL) and MeOH (8 mL) under magnetic agitation (500 rpm) until optical clarity was achieved. The synthetic progression involved the gradual introduction of Solution A into Solution B under vigorous mechanical stirring (800 rpm). The resultant mixture was maintained at ambient conditions (25 ± 2 °C) for 24 h to facilitate ZIF‐8 formation encapsulating iron species. The colloidal product was isolated via high‐speed centrifugation (10 000 × g, 5 min), followed by three sequential washing cycles with anhydrous MeOH to remove residual reactants. Final purification was achieved through vacuum drying (60 °C, 0.1 MPa) for 12 h to yield Fe_2_(CO)_9_@ZIF‐8. The analogous Fe(acac)_2_@ZIF‐8 was synthesized following identical protocols, with Fe_2_(CO)_9_ substituted by Fe(acac)_2_ (16 mg, 0.045 mmol) as the iron precursor.

### Synthesis of Fe_2_‐NCs

The Fe_2_(CO)_9_@ZIF‐8 precursor material was first mechanically pulverized and uniformly distributed within a quartz combustion vessel, which was then positioned at the constant‐temperature zone of a horizontal tubular furnace (GSL‐1700X, Hefei Kejing). A controlled carbonization process was executed under continuous argon flow (99.99% purity, 100 sccm) with the following thermal profile: linear temperature ramping at 5 °C min^−1^ to 950 °C target temperature, 3 h isothermal retention, followed by passive cooling to ambient conditions (25 ± 2 °C). The resulting pyrolyzed composite, designated as Fe_2_‐NCs, was utilized without post‐treatment. Parallel synthesis protocols were implemented for Fe_1_‐NCs and the NCs control groups. Fe(acac)_2_@ZIF‐8 and pristine ZIF‐8 precursors were subjected to identical pyrolysis conditions (950 °C/3 h/Ar) to generate Fe_1_‐NCs and NCs, respectively, maintaining equivalent mass loading and spatial positioning within the reaction chamber.

### Surface Modification of the Prepared Nanoparticles

To optimize biological compatibility, 2 mg DSPE‐PEG‐FA was introduced into 10 mL aqueous dispersion of Fe_2_‐NCs (1 mg mL^−1^ in deionized water). The mixture underwent intense mechanical agitation (800 rpm) followed by 24 h incubation at physiological temperature (37 ± 0.5 °C) to facilitate amphiphilic polymer self‐assembly. Post‐functionalization purification was achieved through three cycles of centrifugal washing (12 000 × g, 10 min) with ultrapure water (18.2 MΩ cm) and subsequent lyophilization (−50 °C, 0.1 Mbar) to obtain PEG@Fe_2_‐NCs. A covalent conjugation system was established by dissolving RhB (5 mg), EDC (10 mg), and NHS (15 mg) in 5 mL PBS (pH 7.4). After 30 min activation in dark conditions (25 °C), 10 mg PEG@Fe_2_‐NCs were introduced into the reaction medium. The suspension was maintained under continuous dark agitation (24 h, 200 rpm) to ensure stable amide bond formation. The resultant RhB‐PEG@Fe_2_‐NCs were isolated through triple PBS washing (0.01 m, pH 7.4) and lyophilized preservation. Identical functionalization protocols were applied to Fe_1_‐NCs and NCs substrates to generate RhB‐PEG@Fe_1_‐NCs and RhB‐PEG@NCs respectively, maintaining equivalent mass ratios and reaction kinetics throughout the process.

### Measurement of OXD‐Like Activity

The catalytic performance evaluation was systematically performed in a 96‐well microplate system under ambient conditions (25 ± 2 °C). Each reaction system contained 220 µL total volume (100 µL chromogenic substrate (0.4 mg mL^−1^ TMB in acetate buffer, pH 4.0), 100 µL reaction buffer (50 mm phosphate buffer, pH 7.0), 20 µL H_2_O_2_ solution (1.3 mm), 20 µL nanomaterial suspension (1 mg mL^−1^)). Time‐dependent spectrophotometric monitoring was conducted at 652 nm wavelength using a multimode microplate reader (SpectraMax i3x, Molecular Devices). Catalytic kinetics were analyzed through kinetic curve fitting using SoftMax Pro 7.0 software, with initial reaction rates calculated from the linear phase of absorbance‐time plots to quantify OXD‐like activity.

### Measurement of CAT‐Like Activity

The CAT‐like catalytic performance of nanozymes was quantitatively assessed through two complementary methodologies. First, a precision dissolved oxygen detection system (JPB‐607A, Shanghai INESA Scientific Instrument) was employed to track molecular oxygen evolution in real time. Reactions were conducted in 0.1 m phosphate buffer (pH 7.4 ± 0.1) under thermostatic control (25.0 ± 0.5 °C). Nanozyme suspensions (1 mg mL^−1^) were introduced into the reaction chamber, followed by H_2_O_2_ substrate addition (final concentration 50 mm). Oxygen saturation levels were recorded at 15 s intervals over 10 min. Subsequently, a commercial catalase activity analysis system (Sigma–Aldrich MAK436) was utilized based on enzymatic H_2_O_2_ decomposition kinetics. The standardized workflow comprised: 1) Freshly prepared 250 mm H_2_O_2_ in ice‐PBS (pH 7.4), 2) Combining 10 µL nanozyme suspension with 40 µL detection buffer; 3) Supplementing 10 µL H_2_O_2_ solution (250 mm); 4) Introducing 450 µL stop reagent post 180 s incubation; 5) Transferring 10 µL reaction mixture to 96‐well plate with 200 µL chromogen, measuring A_240_ after 20 min. Subsequently, Electron spin resonance spectroscopy (Bruker EMXplus, X‐band) was implemented to monitor hydroxyl radical (·OH) dynamics in Fenton‐like systems, containing 100 µM DMPO spin trap, 1 mm Fe^2+^, and 10 mm H_2_O_2_ were analyzed under a nitrogen atmosphere. Characteristic quartet signals (1:2:2:1 intensity ratio) were recorded at g = 2.006 with 1 G modulation amplitude.

### Measurement of SOD‐Like Activity

The O_2_•^−^ scavenging capacity of nanozymes was evaluated through an optimized enzymatic assay based on the McCord‐Fridovich principle, employing WST‐8 (2‐(2‐methoxy‐4‐nitrophenyl)‐3‐(4‐nitrophenyl)‐5 (2,4‐disulfophenyl)‐2H‐tetrazolium sodium salt) as a chromogenic probe instead of conventional nitroblue tetrazolium. The catalytic system comprised xanthine oxidase (XOD, 0.025 U mL^−1^) as the superoxide generator, 300 µm xanthine substrate, 0.1 mM EDTA (ethylenediaminetetraacetic acid) for metal ion chelation, and 100 µm WST‐8 dissolved in 50 mm PBS (pH 7.0 ± 0.1). Reaction kinetics were tracked at 450 nm wavelength using a UV–vis spectrophotometer (Shimadzu UV‐2600i) with 1 nm spectral resolution. The half‐maximal inhibitory concentration (IC_50_), defined as the nanozyme mass concentration required to suppress 50% of WST‐8 oxidation rate, served as the quantitative metric for SOD‐mimetic performance. Control experiments validated assay specificity through parallel measurements with native bovine erythrocyte SOD (Sigma–Aldrich S9697, 3000 U mg^−1^).

### Measurement of GPx‐Like Activity

The GPx‐mimetic catalytic efficiency was evaluated through an enzymatic coupling method employing glutathione reductase. The catalytic process was carried out in 500 µL reaction volumes maintained at physiological temperature (37 °C), comprising a PBS (pH 7.4, 50 mm) supplemented with 1.0 mm EDTA chelating agent. The reaction system contained essential components including 1.0 U mL^−1^ glutathione reductase enzyme, 1.0 mm reduced glutathione (GSH), dimethyl sulfoxide‐dissolved catalytic materials, and 0.25 mm β‐nicotinamide adenine dinucleotide phosphate (NADPH). Following a 3‐min equilibration period, the enzymatic process was triggered by introducing 0.5 mm H_2_O_2_ as an oxidative initiator. Kinetic analysis was performed through continuous spectrophotometric measurement (λ = 340 nm) utilizing a UV–vis spectrophotometric system, with the extinction coefficient (ε) of 6220 M^−1^ cm^−1^ being applied to calculate enzymatic activity based on NADPH oxidation kinetics.

### Theoretical Calculations

The computational investigations were conducted within the framework of density functional theory (DFT) utilizing the Vienna Ab Initio Simulation Package (VASP) integrated with projector augmented wave (PAW) pseudopotentials. Exchange‐correlation interactions were approximated through the Perdew–Burke–Ernzerhof (PBE) functional under the generalized gradient approximation formalism. For the plane wave basis expansion, an energy threshold of 450 eV was implemented to ensure computational precision. To simulate surface environments, periodic boundary conditions were established by creating a 15 angstrom vacuum separation around iron catalytic centers embedded in nitrogen‐enriched carbon matrices. Electron correlation effects, particularly those involving 3d orbital electrons of iron species, were addressed through dispersion‐corrected DFT calculations incorporating Grimme's D3 empirical correction methodology. Reciprocal space sampling was executed using a Monkhorst–Pack grid configuration (2 × 2 × 1) for Brillouin zone integration. Structural relaxation processes and energy computations employed a Gaussian‐type smearing function with 0.05 eV width to enhance numerical stability. The iterative convergence parameters were defined as 10^−5^ eV for total energy differences and 0.01 eV Å^−1^ for residual atomic forces, respectively. Thermodynamic analyses focused on determining Gibbs free energy profiles through systematic evaluation of oxygen dissociation pathways and adsorption–desorption equilibria at distinct iron coordination sites (designated Fe_1_ and Fe_2_). The modeled nanozyme architecture comprised atomic‐scale metal centers coordinated within optimized graphene‐based frameworks, with a characteristic lattice parameter of 16.02 Å determined through comprehensive parametric optimization studies.

### Cell Culture

The ATDC‐5 chondrogenic cell line, procured from Shanghai‐based Cell Verse and subjected to STR profiling verification by Biowing Biotech (Shanghai), was maintained in Dulbecco's modified Eagle's medium (DMEM, glucose concentration 4.5 g L^−1^) enriched with 10% (v/v) heat‐inactivated FBS and antimicrobial agents (penicillin 100 U mL^−1^‐streptomycin 100 µg mL^−1^). Cellular propagation occurred under standardized culture conditions (37 °C, 5% CO_2_ partial pressure, 95% relative humidity) within a controlled atmosphere incubator. Routine maintenance involved complete medium replacement at 48‐h intervals, with subculture procedures initiated upon reaching 80–90% monolayer confluence using enzymatic dissociation protocols. In vitro, considering the sufficient efficacy in eliminating intracellular ROS to effectively protect chondrocytes from oxidative stress‐induced apoptosis, enhance mitochondrial function, and without showing significant cytotoxicity, a concentration of 5 µg mL^−1^ was selected. Besides that, the SOD‐like, CAT‐like, and GPx‐like activities of the nanozyme approach saturation levels at 5 µg mL^−1^, indicating optimal catalytic performance in clearing ROS.

### In Vitro Cytotoxicity Assays

The cytotoxicological assessment of nanozymes was conducted through mitochondrial dehydrogenase activity analysis using a Cell Counting Kit‐8 (CCK‐8, Dojindo Laboratories) colorimetric assay. ATDC‐5 chondrocytes (1 × 10^3^ cells well^−1^) were initially plated in 96‐well culture plates and allowed to adhere under standard culture conditions (37 °C, 5% CO_2_) for 24 h. Following 24‐h exposure to varying concentrations of nanozyme formulations (0–200 µg mL^−1^), 10% (v/v) CCK‐8 solution was introduced to each well. After 30‐min chromogenic reaction at physiological temperature, spectral measurements were performed at 450 nm wavelength using a Bio‐Rad model‐680 microplate reader equipped with wavelength‐specific detection modules. The relative cellular metabolic activity was calculated according to the following Equation (1):

(1) $$\mathrm{Cell}\;\mathrm{viability}\left(\%\right)=\left(A_{\mathrm{sample}}-A_{\mathrm{blank}}\right)/\left(A_{\mathrm{control}}-A_{\mathrm{blank}}\right)\times 100\%$$

where A_control_ represents the optical density of untreated cells cultured in complete medium, A_sample_ denotes the absorbance of nanozyme‐treated groups, and A_blank_ corresponds to cell‐free background values. All experimental data were normalized to the negative control group (0 µg mL^−1^ nanozyme) to determine dose‐dependent cytotoxicity profiles.

### Cellular Uptake of Nanozyme in ATDC‐5 Cells

The cellular internalization dynamics of engineered nanozyme formulations were systematically evaluated through fluorescence colocalization analysis combining nuclear counterstaining and intrinsic nanoparticle fluorescence. Specifically, ATDC‐5 chondrocytes were established as confluent monolayers in 24‐well culture plates through 24‐h incubation under standard growth conditions (37 °C, 5% CO_2_). Subsequently, the cellular models were exposed to RhB‐conjugated PEG‐coated iron nanoclusters (RhB‐PEG@Fe_2_‐NCs) at a concentration of 100 mg mL^−1^ for predetermined intervals. Following nanoparticle exposure, a standardized Hoechst 33342 nuclear counterstaining protocol was implemented by administering 500 µL staining solution per well, followed by 5 min ambient temperature incubation. Three sequential PBS (pH 7.4) washing cycles were rigorously performed to eliminate unbound fluorophores prior to microscopic examination. Spatial correlation between subcellular structures and nanozyme distributions was resolved using a Zeiss LSM900 laser scanning confocal system equipped with airyscan super‐resolution modules. Multichannel image acquisition was conducted with optimized excitation/emission parameters: 405 nm laser (Hoechst 33342) and 552 nm laser (RhB fluorescence), employing appropriate spectral unmixing protocols to prevent signal bleed‐through. Semi‐quantitative colocalization coefficients were calculated using ZEN imaging software to objectively assess nanoparticle internalization efficiency.

### Intracellular Determination of ROS

The oxidative stress dynamics induced by nanozyme activity were assessed through fluorometric detection of intracellular radical species using 2′,7′‐dichlorofluorescein diacetate (DCFH‐DA, Sigma–Aldrich), a cell‐permeable probe that undergoes enzymatic deacetylation and subsequent oxidation to fluorescent DCF in the presence of reactive oxygen intermediates. ATDC‐5 chondrocytes were seeded into six‐well culture vessels (5 × 10^4^ cells well^−1^) and stabilized under standard growth conditions (37 °C, 5% CO_2_) for 24 h. Following medium aspiration, cellular monolayers were administered with catalytic nanozymes (10 µg mL^−1^) in serum‐free DMEM for 4‐h exposure at physiological temperature. Three successive PBS (0.01 m, pH 7.4) rinses were executed to remove residual nanoparticles prior to oxidative challenge. Cellular systems were then subjected to 1‐h H_2_O_2_ (100 µm) stimulation at 37 °C to induce radical generation. DCFH‐DA (10 µm in PBS) was subsequently introduced and allowed to permeate cellular membranes during 60 min incubation under dark conditions. Fluorescence‐activated cell sorting (FACS) analysis was performed on a BD LSR Fortessa flow cytometer equipped with a 488 nm argon laser excitation source, with DCF emission signals quantified through FL‐2 detection channels (530/30 nm bandpass filter). Signal intensities were normalized against fluorescence calibration standards (Spherotech calibration beads) to ensure inter‐experiment consistency.

### Detection of Superoxide Anion Radicals (O_2_•^−^) using DHE

Superoxide anion levels were determined using a Reactive Oxygen Species Assay Kit with dihydroethidium (DHE). Briefly, cells were seeded into black, clear‐bottom 96‐well plates at an appropriate density and cultured overnight to allow attachment. Cells were then subjected to oxidative stress by stimulation with different concentrations of H_2_O_2_, followed by treatment with Fe_1_‐NCs or Fe_2_‐NCs. After 24 h, cells were washed three times with PBS to remove residual compounds. Fresh DHE working solution was added to each well, and the cells were incubated at 37 °C in the dark for 20–30 min. Following incubation, wells were washed once with PBS, and fluorescence was measured using a microplate reader (excitation ≈510 nm, emission ≈580 nm). Each experimental condition was tested in at least triplicate wells.

### Detection of Hydroxyl Radicals (•OH) using HPF

Hydroxyl radical generation was assessed using a Reactive Oxygen Species Assay Kit for hydroxyl radicals (HPF). Cells were plated into black, clear‐bottom 96‐well plates at the appropriate density and incubated overnight. Cells were then stimulated with gradient concentrations of H_2_O_2_ and treated with Fe_1_‐NCs or Fe_2_‐NCs as indicated. After the treatment period (24 h), cells were washed three times with PBS. HPF staining solution was freshly prepared according to the manufacturer's instructions and added to each well. Plates were incubated at 37 °C in the dark for 20–30 min. Following incubation, wells were washed once with PBS, and fluorescence was measured using a microplate reader (excitation ≈490 nm, emission ≈515 nm). All samples were measured in at least triplicate.

### Cell Apoptosis Analysis

Quantitative cytofluorimetric evaluation of programmed cell death was systematically conducted through Annexin V‐FITC/PI dual‐labeling methodology. Chondrocyte populations (1 × 10^5^ cells well^−1^) were initially established in six‐well culture platforms and stabilized under standard incubation parameters (37 °C, 5% CO_2_) for 24‐h adherence. Post‐treatment cellular specimens were harvested through centrifugal separation (300 × g, 5 min) followed by enzymatic dissociation using 0.25% trypsin‐EDTA solution. Sequential PBS washing cycles (pH 7.4, 4 °C) were implemented to ensure complete protease inactivation prior to apoptosis profiling.

Cellular suspensions were reconstituted in calcium‐supplemented binding buffer (10 mm HEPES, 140 mm NaCl, 2.5 mm CaCl_2_, pH 7.4) and dually labeled with Annexin V‐FITC conjugate (5 µL) and propidium iodide (5 µL) through 15‐min dark‐phase incubation at ambient temperature. Post‐staining procedures involved two additional buffer rinses to eliminate unbound fluorophores, followed by final resuspension in 300 µL assay‐specific buffer. Multiparametric apoptosis discrimination was executed on a BD FACS Canto II flow cytometer (BD Biosciences) configured with dual‐laser excitation (488 nm/640 nm), employing standardized threshold settings for viable (Annexin V^−^/PI^‐^), early apoptotic (Annexin V^+^/PI^−^), late apoptotic (Annexin V^+^/PI^+^), and necrotic (Annexin V^−^/PI^+^) population discrimination. Fluorescence compensation matrices were established using single‐stained controls, with subsequent data quantification performed in Flow Jo v10.8 software (BD Life Sciences) incorporating quadrant gating strategies and population.

### Measurement of Mitochondrial Membrane Potential (MMP)

The electrochemical gradient across mitochondrial membranes was quantitatively evaluated through potential‐sensitive fluorometric analysis utilizing JC‐1 (5, 5′, 6, 6′‐tetrachloro‐1, 1′, 3, 3′ tetraethylbenzimidazolylcarbocyanine iodide), a cationic carbocyanine dye exhibiting wavelength‐dependent aggregation states. Cellular systems (1 × 10^5^ cells well^−1^) were established in six‐well culture platforms and maintained under standardized growth conditions (37 °C, 5% CO_2_) for 24 h monolayer formation. Post‐treatment specimens were collected via enzymatic detachment (0.25% trypsin‐EDTA) and reconstituted in 500 µL serum‐free DMEM (Gibco).

The JC‐1 working solution (5 µg mL^−1^ in DMEM) was introduced in equal volume (1:1 v/v) to cellular suspensions, followed by 20‐min dark‐phase incubation at physiological temperature. Subsequent to dual centrifugation‐wash cycles (300 × g, 5 min) with iced assay‐specific buffer (150 mm NaCl, 5 mm KCl, 1 mm MgCl_2_, 2 mm CaCl_2_, 10 mm HEPES, pH 7.4), labeled cells were resuspended in 300 µL fresh buffer for cytofluorimetric analysis. Membrane polarization dynamics were resolved using a BD FACS Canto II flow cytometer (BD Biosciences) conjugated with dual‐laser excitation (488 nm/640 nm), implementing optimized optical configurations: 1. JC‐1 monomers: FL‐1 channel (530/30 nm); 2. J‐aggregates: FL‐2 channel (585/42 nm). Fluorescence intensity ratios (FL‐2/FL‐1) were computed using Flow Jo v10.8 software (BD Life Sciences) with compensation matrices established through single‐stained controls. Mitochondrial depolarization indices were expressed as percentage reduction in aggregate/monomer fluorescence ratios relative to untreated control populations.

### Immunofluorescence Staining Analysis

Immunofluorescence characterization of chondrocyte specimens was performed according to standardized immunostaining protocols. Cellular systems were established in µ‐Dish 35 mm high‐glass bottom confocal culture vessels (Ibidi GmbH) and subjected to experimental protocols as previously described. Following 24‐h maintenance under standard culture parameters (37 °C, 5% CO_2_), biological specimens were fixed in 4% paraformaldehyde (PFA) solution for 15 min at ambient temperature. Non specific binding sites were saturated through 60‐min exposure to blocking buffer containing 5% (w/v) bovine serum albumin (BSA, Sigma–Aldrich) in PBS. Primary antibody incubation was conducted at 4 °C for 16 h using anti‐target protein IgG (1:200 dilution in PBS/0.1% BSA). Subsequent to triple washing cycles with phosphate‐buffered saline containing 0.1% Tween 20 (PBST), specimens were probed with Alexa Fluor 488‐conjugated secondary antibodies (1:500 dilution, Invitrogen) during 2‐h dark‐phase incubation at 25 °C. Nuclear architecture was delineated through 5‐min counterstaining with 4′,6 diamidino‐2‐phenylindole (DAPI, 1 µg mL^−1^, Thermo Fisher Scientific). For histological evaluation, cryosectioned articular tissues (7 µm thickness) were mounted on poly‐L‐lysine coated slides (Thermo Scientific). Following xylene‐based dewaxing and graded ethanol rehydration processes, tissue sections underwent parallel immunostaining procedures as described for in vitro models. Fluorescence quantification was executed using Image J v1.48 software (National Institutes of Health) with standardized parameters.

### Western Blot Analysis

Cellular protein specimens were isolated from ATDC‐5 chondrocytes through ultrasonication in RIPA buffer (25 mm Tris‐HCl pH 7.6, 150 mm NaCl, 1% NP‐40, 1% sodium deoxycholate, 0.1% SDS) containing protease inhibitor cocktail (1 mm PMSF, 10 µg mL^−1^ leupeptin). The lysate underwent refrigerated centrifugation (12 000 × g, 20 min, 4 °C) to pellet insoluble debris, with the clarified supernatant subsequently subjected to bicinchoninic acid (BCA) quantification (PierceTM BCA Assay Kit, Thermo Scientific) for precise normalization of protein loads. Electrophoretic separation was conducted in 12% SDS‐polyacrylamide gels under constant voltage (80 V stacking/120 V resolving) using Tris‐glycine running buffer (25 mm Tris, 192 mm glycine, 0.1% SDS, pH 8.3). Resolved proteins were electrotransferred (100 V, 60 min) onto 0.45 µm nitrocellulose membranes (Bio‐Rad) in Towbin transfer buffer (25 mm Tris, 192 mm glycine, 20% MeOH). Membranes were saturated with 5% (w/v) non‐fat dried milk in TBST (20 mm Tris, 150 mm NaCl, 0.1% Tween 20, pH 7.6) for 2‐h blocking prior to immunoprobing. Membranes were hybridized with primary antibodies in blocking buffer at 4 °C for 16 h, followed by three 10‐min TBST washes. Secondary detection employed HRP‐conjugated anti‐rabbit/mouse IgG (1:5000, Cell Signaling Technology) with 1‐h room temperature incubation. Chemiluminescent signals were developed using ECL Prime substrate (Merck Millipore) and captured on an Amersham Imager 680 (GE Healthcare). Band densitometry was quantified in Image J v1.53e (NIH) with local background subtraction and normalized against GAPDH loading controls.

### qRT‐PCR Analysis

RNA isolation from treated chondrocytes was performed with the EZ Press RNA Purification System (Ezbioscience, RE‐102) according to established protocols. Nucleic acid integrity was verified through spectrophotometric quantification using a NanoDrop ND‐2000 system (Thermo Fisher Scientific), with purity criteria set at A_260_/A_280_ ratios of 1.8:2.1. Custom‐designed primer pairs (Table S6, Supporting Information, synthesized by Sangon Biotech) were engineered following NCBI Primer‐BLAST specifications with T_m_ values optimized at 60 ± 2 °C. Reverse transcription was conducted using Hyperscript III Reverse Transcriptase Supermix (Enxyartisan, RT‐2017K) under thermal cycling parameters: 25 °C for 5 min, 50 °C for 15 min, 85 °C for 5 min.

Quantitative reverse transcription PCR (qRT‐PCR) amplification cycles were performed on an ABI Quant Studio 6 Flex real‐time PCR system (Applied Biosystems) with SYBR Green‐based master mix (Enxyartisan, QP‐3001). Reaction parameters included initial denaturation at 95 °C for 3 min, followed by 40 cycles of 95 °C for 10 s and 60 °C for 30 s, concluding with melt curve analysis (65–95 °C, 0.5 °C increment/5 s). Cycle threshold (Ct) values were normalized against β‐actin endogenous control.

### Transcriptomics (RNA‐Sequencing and Analysis)

Transcriptomic profiling was conducted through next‐generation sequencing services provided by Aksomics Corporation (Shanghai, China). Chondrocyte specimens were isolated from murine models via TRIzol‐based RNA extraction protocol (Invitrogen), involving chloroform phase separation followed by isopropanol precipitation. Experimental cohorts comprised three distinct groups: 1) Non‐stimulated chondrocytes (NSC) ‐ physiological baseline control; 2) Oxidative stress model (H_2_O_2_) ‐ 200 µm H_2_O_2_ stimulation; 3) Therapeutic intervention (H_2_O_2_ + Fe_2_‐NCs) – H_2_O_2_ + 50 µg mL^−1^ iron‐based nanozymes. RNA integrity was verified through Bioanalyzer 2100 assessment (RIN ≥8.0) prior to poly‐A tail enrichment using oligo (dT) magnetic beads. Strand‐specific libraries were prepared with KAPA Stranded mRNA‐Seq Kit (Kapa Biosystems) following Illumina‐compatible protocols. High‐throughput sequencing was executed on NovaSeq 6000 platforms (Illumina) in 150 bp paired‐end mode, with triplicate biological replicates per group ensuring statistical robustness. Bioinformatic processing involved: Raw read alignment via HISAT2 (v2.2.1) against mm10 reference genome; Transcript quantification using feature Counts (v2.0.1); Differential expression analysis through limma‐voom pipeline (R v4.2.1). Cross‐cohort comparison strategy: OA pathogenesis: NSC vs H_2_O_2_ (FDR<0.05, log_2_FC≥|0.3|); Therapeutic mechanism: H_2_O_2_ vs H_2_O_2_ + Fe_2_‐NCs (FDR<0.05, log_2_FC≥|0.3|). Functional annotation utilizing: GO term enrichment analysis (cluster Profiler v4.4.4); KEGG pathway annotation (KOBAS v3.0); Heatmap visualization (Complex Heat map v2.12.1).

### Animal Experiment

Female C57BL/6J mice (*n* = 32, 10‐week‐old, 18–20 g body weight) were sourced from Beijing Vital River Laboratory Animal Technology Co., Ltd. (SPF‐grade, certification No. SCXK‐2022‐0012). Murine subjects were acclimatized in IVC isolator cages within an AAALAC‐accredited vivarium (temperature 22–24 °C, humidity 55±5%, 12:12 h photoperiod) with autoclaved chow and acidified water ad libitum. All experimental protocols were approved by the Institutional Animal Care and Use Committee of Fudan University Pudong Medical Center (IACUC‐20230920‐02) following ARRIVE 2.0 guidelines. As delineated in Figure 8a, osteoarthritis modeling was achieved through unilateral right‐knee anterior cruciate ligament transection (ACLT) surgical intervention under 2% isoflurane anesthesia. Postoperative analgesia was maintained with buprenorphine SR (1.0 mg kg^−1^) for 72 h. Following a 7‐day recovery period, animals were randomized into four experimental cohorts: 1) Sham group, 2) ACLT group, 3) Fe_1_‐NCs + ACLT group, and 4) Fe_2_‐NCs + ACLT group. Terminal procedures were conducted at 8‐week post‐interval following the CO_2_ asphyxiation protocol. Synovial joints were harvested for subsequent micro‐CT and histomorphometric analyses. In vivo, considering the significant inhibition of chondrocyte apoptosis, reduction of oxidative stress‐induced cartilage damage, promotion of cartilage layer structure and tissue integrity recovery, and maintenance of joint gap width, 10 µL of 2 mg mL^−1^ nanozyme solution was adopted for intra‐articular injection without showing any biological safety risks.

### In Vivo Biosafety and Toxicity Evaluation

To systematically assess the biological safety and potential toxicity of the nanozymes, healthy male Sprague‐Dawley (SD) rats were employed as experimental subjects. Prior to the initiation of the experiment, the rats underwent an adaptive feeding period to ensure optimal physiological conditions. Throughout the experimental procedure, varying concentrations of nanozymes were administered via lateral ventricle injection to elucidate their effects on the central nervous system. Concurrently, an equal volume of 0.9% sodium chloride solution was injected to establish a control group. Rigorous controls were implemented regarding the timing and dosage of treatments across all groups to uphold the reliability of the experimental outcomes. Following the completion of the administration, the rats were euthanized in accordance with ethical guidelines, and subsequent dissection was performed. The principal organs harvested included the brain, heart, liver, spleen, lungs, and kidneys, selected based on their critical roles within the organism and their representative responses to exogenous substances. The excised organs were subjected to standard histological processing protocols, encompassing paraffin embedding, sectioning, and hematoxylin‐eosin (HE) staining. Through meticulous examination of HE‐stained tissue sections, morphological alterations across various organs induced by the nanozymes were analyzed, providing a comprehensive evaluation of their biological safety and toxicity profiles.

### Microcomputed Tomography (Micro–CT) Evaluation

The high‐resolution micro‐CT scanner Skyscan 1276 (Bruker, Germany) was employed to perform detailed imaging of the knee joints of each mouse, thereby obtaining high‐precision imaging data of their internal structures. The scanning process ensured comprehensive coverage of all joint components, resulting in high‐resolution 3D images that lay the groundwork for subsequent analyses. Upon completion of the scanning, the original images were reconstructed using the 3D reconstruction software NRecon (version 1.7.4.2, Bruker, Germany) to delineate the Regions of Interest (ROIs). This step not only enhanced the accuracy of data processing but also established a solid foundation for further investigation. For the analysis of the selected ROIs, CT Analyzer (version 1.20.3.0, Bruker, Germany) software was utilized to maintain consistent evaluation parameters. This software computed several critical parameters, including total tissue volume (TV), bone volume (BV), subchondral bone plate thickness, and osteophyte size. These parameters are essential for elucidating the OA progression and its associated pathological changes. To ensure the objectivity and accuracy of the analysis results, two professional radiologists employed a blinded methodology during the imaging evaluation, independently assessing the severity of osteoarthritis and the maturity level of osteophytes. The double‐blind evaluation approach significantly mitigated potential subjective biases from the evaluators, thereby enhancing the reliability and validity of the findings.

### Histological Staining

In this study, mouse knee joint tissues were collected for detailed histological evaluation and analysis. The knee joint tissues were first fixed in 4% paraformaldehyde at room temperature for 1 week, with the entire process conducted in the absence of light to prevent any potential effects from illumination on the tissues. After fixation, the tissue samples were transferred to a 10% ethylenediaminetetraacetic acid (EDTA) solution for decalcification, which may last up to one month to effectively remove bone mineral content while preserving the integrity of the cellular structure. Upon completion of decalcification, the tissue samples were embedded in paraffin for subsequent sectioning. The thickness of the embedded tissue sections was set at 7 µm, which is suitable for further microscopic observation and analysis. Following sectioning, the samples were stained using the hematoxylin and eosin (H&E) staining to observe the fine morphological structures, including cell nuclei, cytoplasm, and the overall morphological characteristics of the tissue. Additionally, the Safranin O/Fast Green staining technique was employed to evaluate the distribution of glycosaminoglycans (GAGs) within the knee joint tissues, which was critical for understanding the health status of articular cartilage. By combining these two staining techniques, a more comprehensive assessment of the morphological changes and functional status of the cartilage was conducted. To quantify the degree of cartilage degeneration, 3D computed tomography (3D‐CT) imaging technology was integrated with the results from histological staining, using the Osteoarthritis Research Society International (OARSI) scoring system to assess cartilage degeneration. The OARSI scoring system is a widely recognized standard that effectively evaluates the degeneration of cartilage in osteoarthritis, considering multiple aspects such as cartilage thickness, structural integrity, and cellular viability.

### Ethical Statement

All animal experiments and procedures, including euthanasia, were conducted in accordance with the guidelines approved by the Animal Experiment Ethics Committee of Fudan University Pudong Hospital (approval number: 20230920‐2). The study also received review and approval from the hospital's animal welfare and ethics committee. All animal experiments were conducted in accordance with the ARRIVE guidelines.

### Statistical Analysis

Experimental procedures were conducted in triplicate with independent technical replicates to ensure methodological robustness. Quantitative data are expressed as mean values with standard error, where the specific sample number (n) for each dataset is explicitly annotated in the corresponding Figure captions. Inter‐group comparisons were statistically evaluated through two‐tailed unpaired Student's *t*‐test (assuming unequal variances) using SPSS Statistics v27, with significance thresholds defined at α = 0.05 (*p* <0.05 considered statistically significant, ^*^ *p*<0.05; ^**^ *p*<0.01; ^***^ *p*<0.001). Biological triplicates (n≥3) were maintained across all experimental conditions unless otherwise specified.

## Conflict of Interest

The authors declare no conflict of interest.

## Supporting information



Supporting Information

## Data Availability

The data that support the findings of this study are available from the corresponding author upon reasonable request.
