# Supplementary material for: Constructing Dual‐Atomic Fe─Fe Sites Nanozyme for Targeted Osteoarthritis Therapy through Mitigating Oxidative Stress and Cartilage Degeneration
Source: Adv Sci (Weinh). 2025 Oct 20;13(1):e08073. doi: 10.1002/advs.202508073 (PMC12767132; doi:10.1002/advs.202508073)
Supplement: Supplementary file 1 — Supporting Information [file ADVS-13-e08073-s001.docx]

**Supporting Information**

**Constructing Dual-Atomic Fe-Fe Sites Nanozyme for Targeted Osteoarthritis Therapy through Mitigating Oxidative Stress and Cartilage Degeneration**

*Ting Ying^a,#^, Qi Wang^b,#^, Dejian Li^b^, Yao Wang^c,*^, Pengfang Zhang^d^, Chengqing Yi^b,*^, Rui Zhu^a*^*

T. Ying, Prof. R. Zhu

Shanghai Yangzhi Rehabilitation Hospital (Shanghai Sunshine Rehabilitation Center), School of Medicine, Tongji University, Shanghai 200092. China

Email: [zhurui08@hotmail.com](mailto:zhurui08@hotmail.com)

Q. Wang, Dr. D.J. Li, Prof. C.Q. Yi

Department of Orthopedics, Shanghai Pudong Hospital, Fudan University Pudong Medical Center, Shanghai 201300, China.

E-mail: [ycq3000@163.com](mailto:ycq3000@163.com)

Prof. Y. Wang

Key Laboratory of Synthetic and Biological Colloids, Ministry of Education, School of Chemical and Material Engineering, Jiangnan University, Wuxi 214122, China.

Email: [wangyao@jiangnan.edu.cn](mailto:wangyao@jiangnan.edu.cn)

Dr. P.F. Zhang

Shandong Provincial Key Laboratory of Chemical Energy Storage and Novel Cell Technology, Liaocheng University, Liaocheng, 252000 China

^#^ These authors contributed equally to this work.

**Materials**

Zinc nitrate hexahydrate (Zn(NO_3_)_2_*6H_2_O, 99% purity) was sourced from Shanghai Runjie Chemical Reagent Co., Ltd. The organic ligand 2-methylimidazole (2-mIM, 99%) along with anhydrous methanol (MeOH, 99.9%) and ethanol (EtOH, 99.9%) were acquired from Titan Scientific Co., Ltd. (Shanghai, China). Ferric acetylacetonate (Fe(acac)_2_, ≥98.0%), N,N-dimethylformamide (DMF, 99%), and ethylene glycol (EG, 99.9%) were supplied by Sinopharm Chemical Reagent Co., Ltd. Diiron nonacarbonyl (Fe_2_(CO)_9_, ≥97.0%) and hydrogen peroxide (H_2_O_2_, 30% aqueous solution) ) were procured from Macklin Biochemical Co., Ltd. Specialized biochemical reagents including 3,3’,5,5’-tetramethylbenzidine (TMB), rhodamine B (RhB), 1-(3-dimethylaminopropyl)-3-ethylcarbodiimide hydrochloride (EDC)/N hydroxysuccinimide (NHS) coupling agents, and folate-conjugated DSPE-PEG phospholipid (DSPE-PEG FA) were provided by Shanghai Aladdin Biochemical Technology Co., Ltd. Cell culture supplements such as DMEM/F12 medium, fetal bovine serum (FBS), and penicillin-streptomycin were purchased from Gibco (USA). Analytical-grade detection kits—CCK-8 cell viability assay, DCFH-DA ROS detection, DHE ROS detection, HPF ROS detection, JC-1 mitochondrial membrane potential analysis, and MitoTracker Red—were obtained from Beyotime Institute of Biotechnology (China). Additional reagents including WST-8 tetrazolium salt, SOD activity assay kit, Calcein/PI live/dead staining solution, DAPI nuclear dye, and H&E staining kit were sourced from the same supplier. Antibody: collagen type II antibody, matrix metallopeptidase 13 (MMP13) antibody, cyclooxygenase 2 (COX-2) antibody, NF-κB antibody, phospho-NF-κB antibody, NADPH oxidase 4 (NOX4), cytochrome c oxidase subunit IV (COXIV), GAPDH antibody and HRP-conjugated goat anti-rabbit/mouse IgG (H+L) secondary antibody were purchased from Proteintech Group (Wuhan, Hubei, China). Goat anti-rabbit/mouse IgG H&L (alexa fluor® 488/594) were purchased from Abcam (Cambridge, UK). All chemicals and solvents were utilized as received without additional purification. Ultrapure water (18.2 MΩ*cm resistivity) was generated through a Millipore filtration system for experimental use.

**Characterization**

The morphological and structural properties of the synthesized nanomaterials (NCs, Fe_1_-NCs, and Fe_2_-NCs) were systematically investigated using advanced characterization techniques. Field-emission scanning electron microscopy (FE-SEM, Hitachi S-4800) operated at 5 kV was first employed to examine surface topography. Prior to imaging, all specimens were sputter-coated with a 5 nm gold layer under Ar atmosphere to enhance conductivity. Nanoparticle size distribution was quantified via Image J software (National Institutes of Health, USA) through statistical analysis of ≥20 distinct regions across multiple SEM micrographs. For higher-resolution structural insights, transmission electron microscopy (TEM) was conducted using a Hitachi HT7700 instrument at 100 kV acceleration voltage. Specimens for TEM were prepared by drop-casting ethanol-dispersed nanostructures onto carbon-coated copper grids followed by ambient drying. Atomic-scale imaging was achieved through high-resolution TEM (HRTEM) and high-angle annular dark-field scanning TEM (HAADF-STEM) using a JEOL JEM-2100F (200 kV) and spherical aberration-corrected JEOL JEM-ARM200F (300 kV), respectively. Elemental composition mapping was performed via energy-dispersive X-ray spectroscopy (EDS) coupled with SEM. Crystalline phase identification was carried out by X-ray diffraction (XRD, Rigaku D/max-2200) with Cu Kα radiation (λ = 1.5406 Å, 40 kV, 200 mA), scanning 2θ =5-80° at 2 °min^-1^ with 0.02° step resolution. Functional group analysis was conducted using Fourier-transform infrared spectroscopy (FTIR, PerkinElmer Frontier) across 4000-400 cm^-1^ spectral range. Surface chemical states were probed by X-ray photoelectron spectroscopy (XPS, Thermo Scientific PHI5700 ESCA, USA) with C 1s (284.6 eV) as charge reference. Iron content quantification was achieved through inductively coupled plasma optical emission spectroscopy (ICP-OES, Thermo iCAP 7400) following acid digestion (HCl:HNO_3_ =3:1, v/v). Adsorption-desorption properties including specific surface area and pore size distribution were determined from N_2_ adsorption-desorption isotherms at 77 K (Micromeritics ASAP 2020), applying Brunauer-Emmett-Teller (BET) and Barrett Joyner-Halenda (BJH) models respectively. Raman spectroscopy (Horiba LabRAM HR Evolution) with 532 nm excitation laser was utilized for crystallinity assessment. Synchrotron-based X-ray absorption near edge structure (XANES) at C K-edge and N K-edge was performed at NSRL BL12B beamline (Hefei, China). Samples were homogenized with cellulose to ~1000 ppm metal concentration and mounted on carbon tape. Extended X-ray absorption fine structure (EXAFS) at Fe K-edge was acquired at SSRF BL11B station (2.5 GeV, 250 mA), with data processed using Athena software.

**Supplementary Figures and Tables**


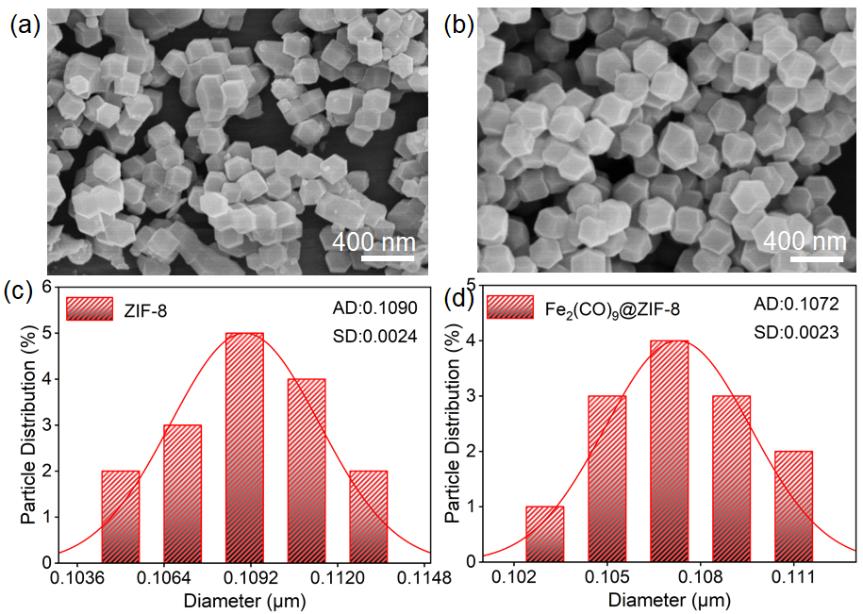


**Figure S1**. SEM images of the (a) ZIF-8, (b) Fe_2_(CO)_9_@ZIF-8. Corresponding the particle size distribution of (c) ZIF-8, (d) Fe_2_(CO)_9_@ZIF-8.


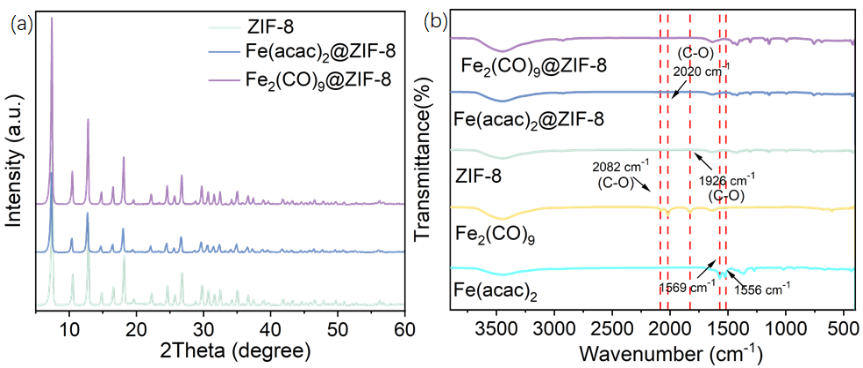


**Figure S2.** (a) XRD patterns of ZIF-8, Fe(acac)_2_@ZIF-8 and Fe_2_(CO)_9_@ZIF-8. (b) FTIR spectra of Fe(acac)_2_, Fe_2_(CO)_9_, ZIF-8, Fe(acac)_2_@ZIF-8, and Fe_2_(CO)_9_@ZIF-8.

**Note**: The crystalline phases of the ZIF-8, Fe(acac)_2_@ZIF-8, and Fe_2_(CO)_9_@ZIF-8 were investigated by the powder X-ray diffraction (XRD) technology. As exhibited in **Figure S2a** (**Supporting Information**), the obvious characteristic diffraction peaks centered at 2θ of 7.29°, 10.44°, 12.83° and 18.10°are indexed to the (110), (200), (211), and (222) planes in a typical ZIF-8 structure, respectively. The Fourier transform infrared spectra (FTIR) was performed in the wavenumber range of 4000-400 cm^-1^. As shown in Figure S2b (Supporting Information), the characteristic peaks at 2082, 2020, and 1926 cm^-1^ in the prepared samples (Fe(acac)_2_@ZIF-8, and Fe_2_(CO)_9_@ZIF-8) were assigned to the *v*_C-O_ vibrations, indicating that Fe(acac)_2_ and Fe_2_(CO)_9_ have been successfully encapsulated in the cavity of ZIF-8, in which the Fe(acac)_2_@ZIF-8, and Fe_2_(CO)_9_@ZIF-8 shows the same responding signals with ZIF-8.


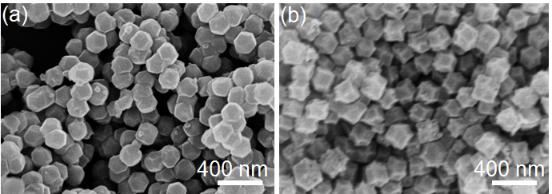


**Figure S3**. SEM images of the (a) NCs, (b) Fe_2_-NCs.


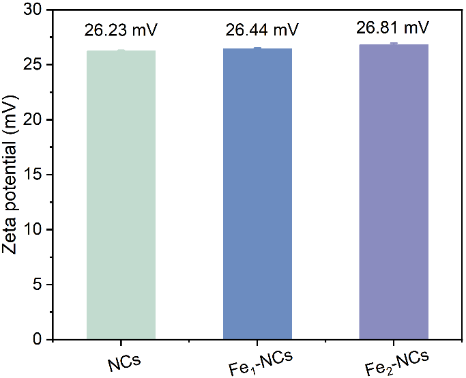


**Figure S4.** Zeta potential of the prepared nanozymes (NCs, Fe_1_-NCs and Fe_2_-NCs).


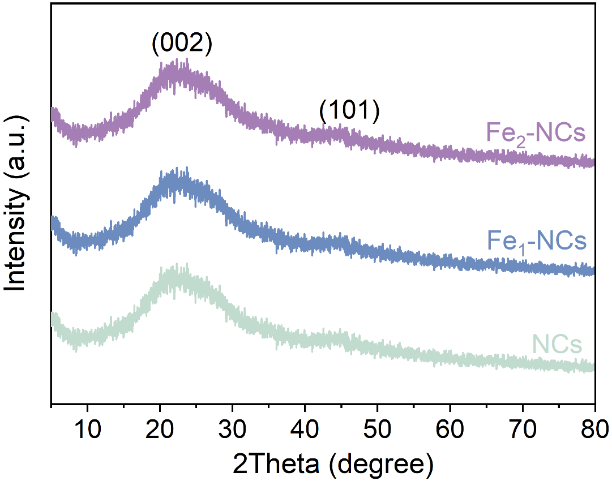


**Figure S5.** XRD patterns of NCs, Fe_1_-NCs and Fe_2_-NCs.


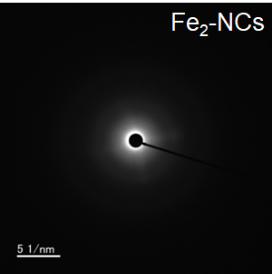


**Figure S6.** The selected-area electron diffraction (SAED) patterns of Fe_2_-NCs.


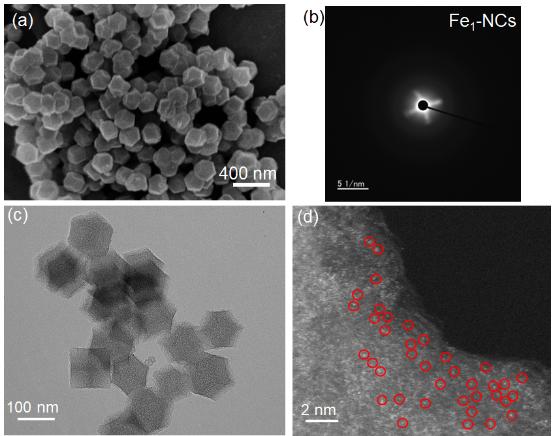


**Figure S7**. (a) SEM, (b) SAED, (c) TEM, (d) HAADF-STEM images of the Fe_1_-NCs.


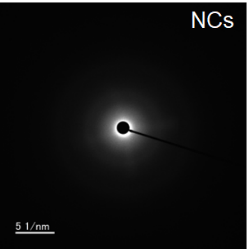


**Figure S8**. SAED images of the NCs.


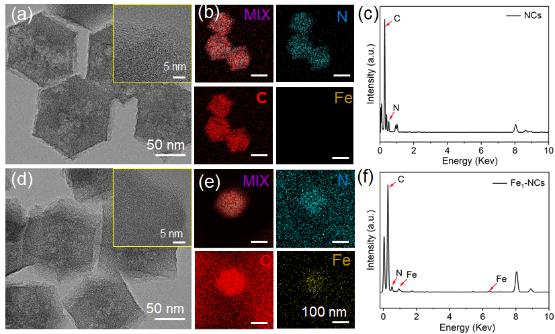


**Figure S9**. (a, d) TEM images, and corresponding HRTEM magnification images inserted in each TEM image of NCs and Fe_1_-NCs, respectively. (b, e) Elemental mapping, and (c, f) Corresponding, the intensity of element contents of NCs and Fe_1_-NCs, respectively.


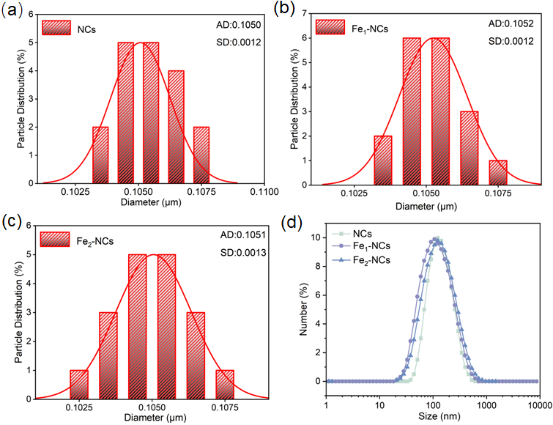


**Figure S10**. The Corresponding the particle size distribution of (a) NCs, (b) Fe_1_-NCs (c) Fe_2_-NCs. (d) Hydration size distribution of the prepared nanozymes by DLS.


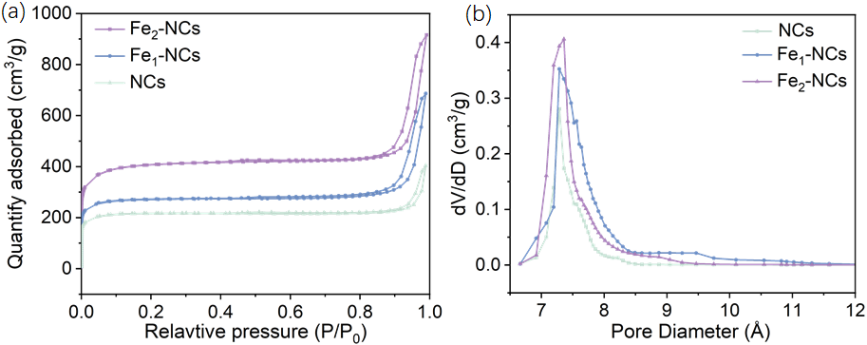


**Figure S11.** Nitrogen adsorption-desorption isotherm (A) and pore size distribution (B) of the NCs, Fe_1_-NCs and Fe_2_-NCs.

**
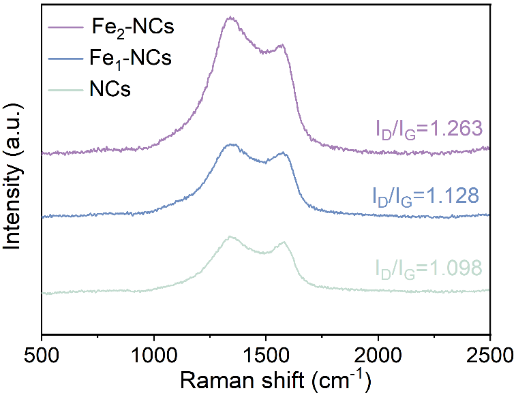
**

**Figure S12.** Raman spectra of NCs, Fe_1_-NCs and Fe_2_-NCs, respectively.


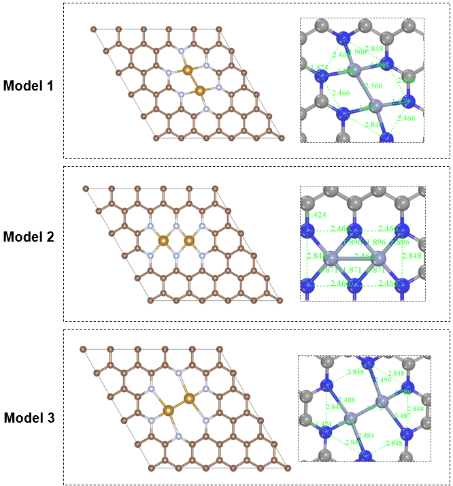


**Figure S13.** Several Fe_2_-NCs models based on the experiment results.


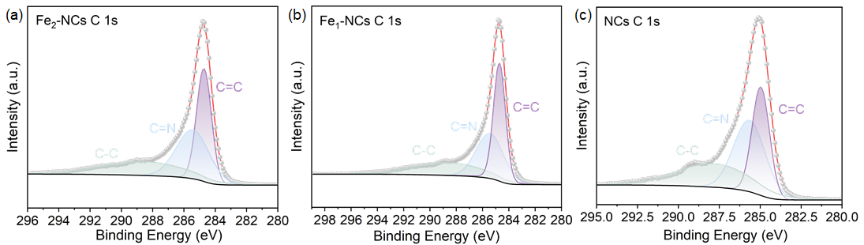


**Figure S14.** C 1s XPS spectrum of Fe_2_-NCs, Fe_1_-NCs and NCs, respectively.


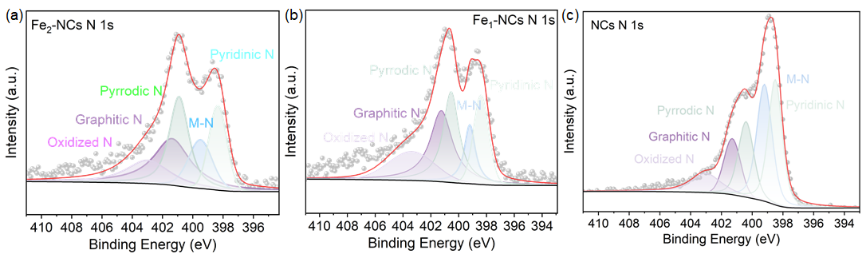


**Figure S15.** N 1s XPS spectrum of Fe_2_-NCs, Fe_1_-NCs and NCs, respectively.


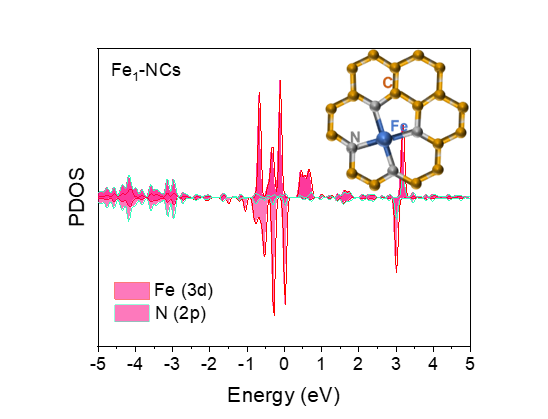


**Figure S16**. The PDOS of Fe_1_-NCs models.


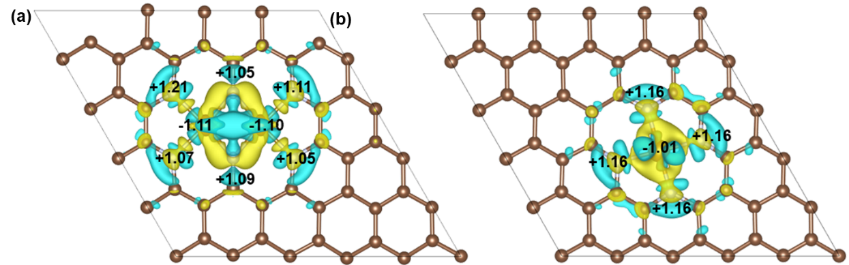


**Figure S17**. Three-dimensional charge density difference of (a) Fe_2_-NCs and (b) Fe_1_-NCs. The iso-value is 0.003e/Å^3^. (yellow: electro-accumulation, cyan: electron depletion)


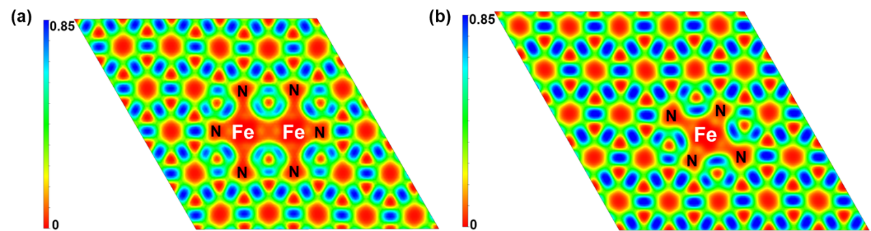


**Figure S18**. Two-dimensional projection of charge density contour of (a) Fe_2_-NCs and (b) Fe_1_-NCs.


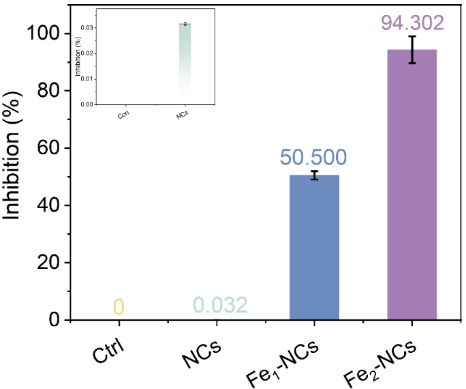


**Figure S19.** SOD-like activities of the prepared nanozymes (NCs, Fe_1_-NCs, and Fe_2_-NCs) (concentration: 100 mg/L).


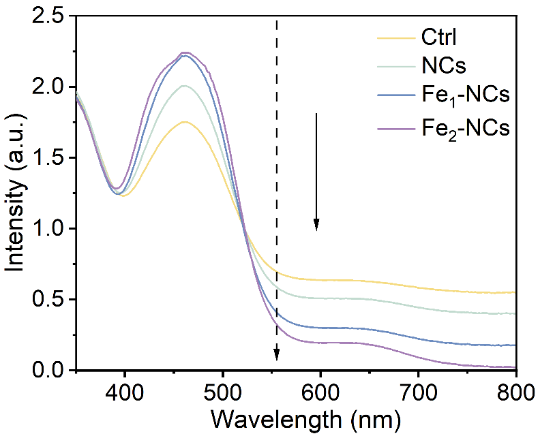


**Figure S20.** UV-vis spectra of reaction solution with the different prepared nanozymes after reaction (concentration: 100 mg/L).


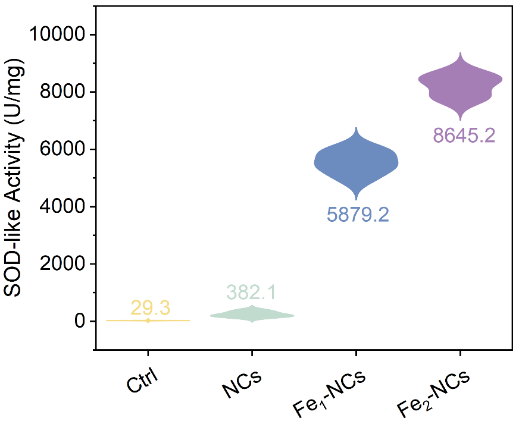


**Figure S21.** Quantification of SOD-like activities of the synthesized nanozymes (NCs, Fe_1_-NCs, and Fe_2_-NCs).


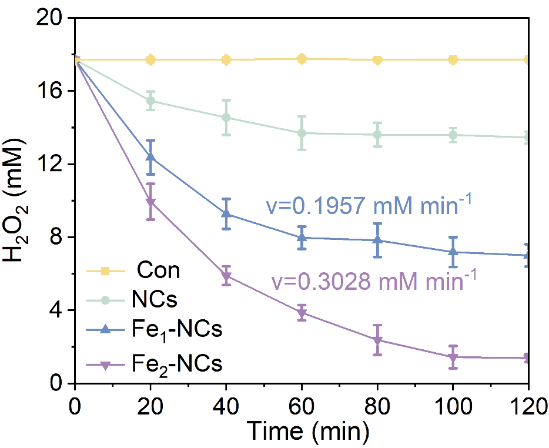


**Figure S22.** Time-dependent absorbance of H_2_O_2_ at 240 nm in the presence of the synthesized nanozymes.


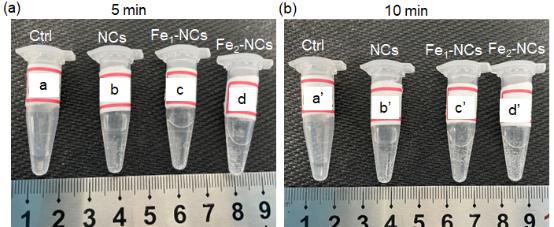


**Figure S23.** The digital images of the O_2_ generation ability of the prepared samples (Ctrl (a, a’), NCs (b, b’), Fe_1_-NCs (c, c’) and Fe_2_-NCs (d, d’)) in different reaction time. The concentration of NCs, Fe_1_-NCs and Fe_2_-NCs was 10 µg/mL. The concentration of H_2_O_2_ was 0.1 M.


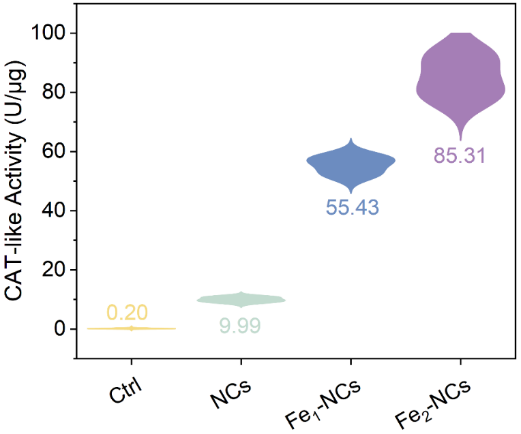


**Figure S24.** Quantification of CAT-like activities of the synthesized nanozymes (NCs, Fe_1_-NCs, and Fe_2_-NCs).


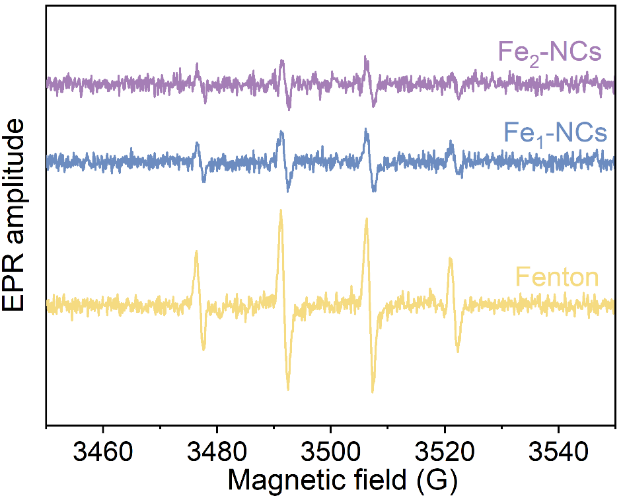


**Figure S25.** Electron paramagnetic resonance (EPR) signal of •OH radical (blank line); Change in DMPO/•OH EPR signal upon addition of Fe_2_-NCs (purple line) and Fe_1_-NCs (blue line).


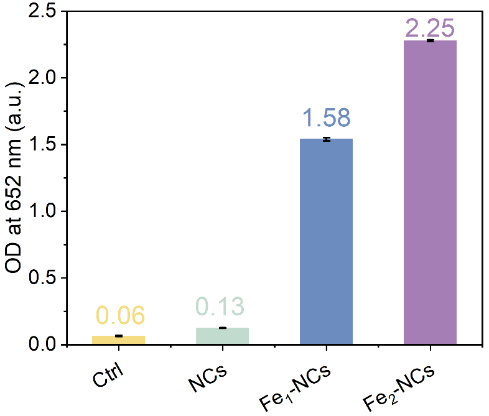


**Figure S26.** OXD-like activities of the prepared nanozymes.


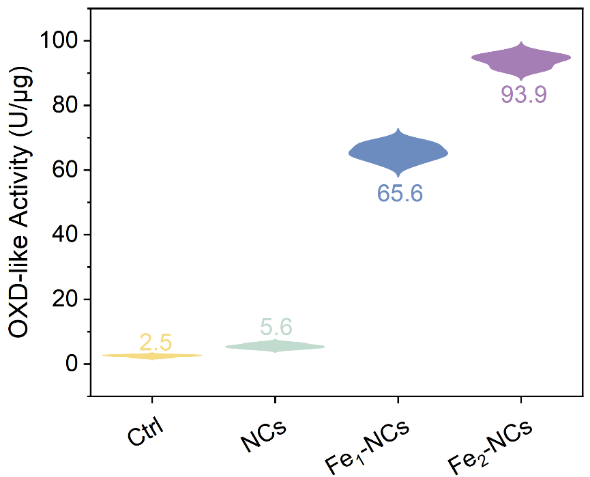


**Figure S27.** Quantification of OXD-like activities of the prepared nanozymes.


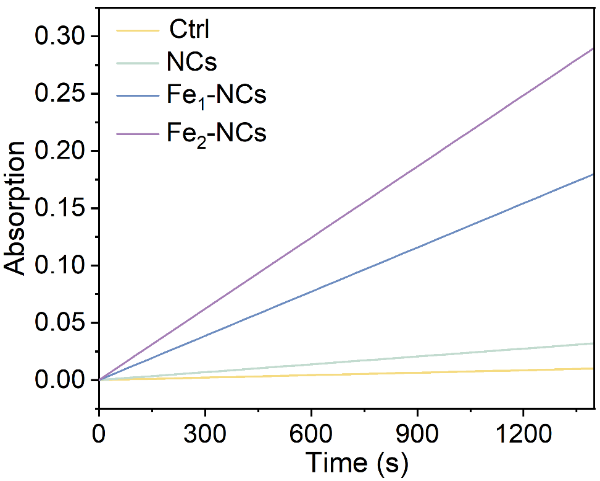


**Figure S28.** Time-dependent absorbance changes of TMB at 652 nm. Initial concentration of TMB was 1 mM.


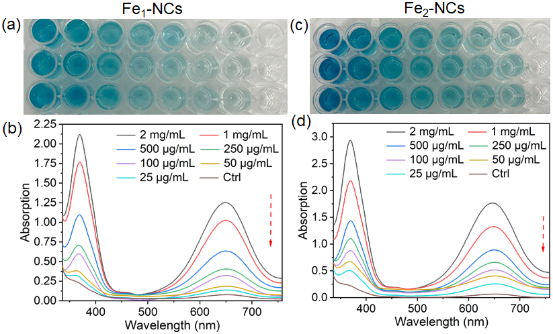


**Figure S29.** Color changes after incubation of (a) Fe_1_-NCs and (c) Fe_2_-NCs with different concentrations of TMB (25 µg/mL, 50 µg/mL, 100 µg/mL, 250 µg/mL, 500 µg/mL, 1 mg/mL and 2 mg/mL). (b, d) Catalytic activity was measured using a TMB assay kit. After Fe_1_-NCs (b) and Fe_2_-NCs (d) catalyzed the TMB to the blue product with a maximum absorption wavelength from 330 to780 nm.


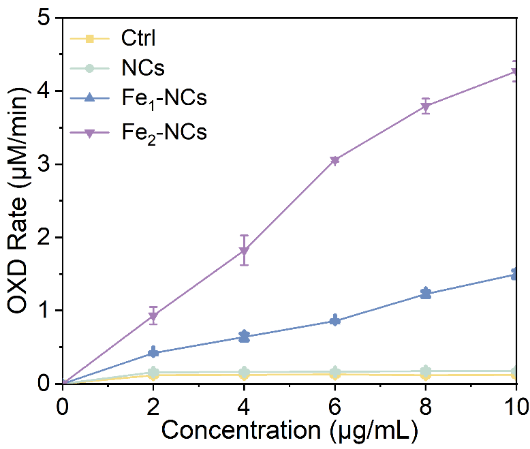


**Figure S30.** OXD reaction rates of the synthesized nanozymes (NCs, Fe_1_-NCs, and Fe_2_-NCs) at different concentration of TMB.


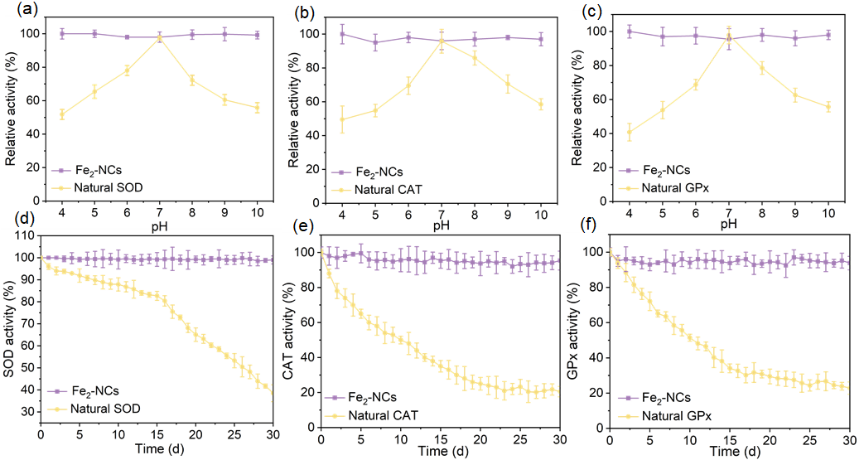


**Figure S31.** (a-c) A comparison of the SOD-, CAT- and GPx-mimicking long-term activity of Fe_2_-NCs with natural SOD (a), CAT (b) and GPx (c). (d, f) pH stability of SOD-mimicking (d), CAT-mimicking (e) and GPx-mimicking (f) activities.


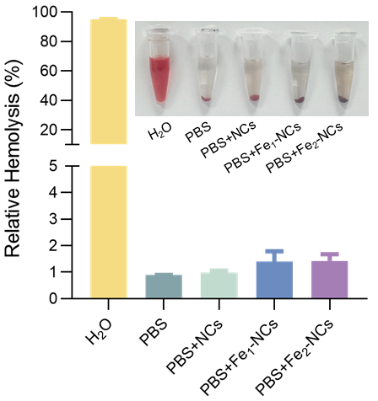


**Figure S32.** Hemolysis rate of erythrocytes in each group after 4 h of incubation.

**Note**: Distilled water (H_2_O) and phosphate buffer solution (PBS) were used as positive and negative controls, respectively, since red blood cells undergo hemolysis when exposed to H_2_O due to osmotic imbalance, while PBS does not induce hemolysis.


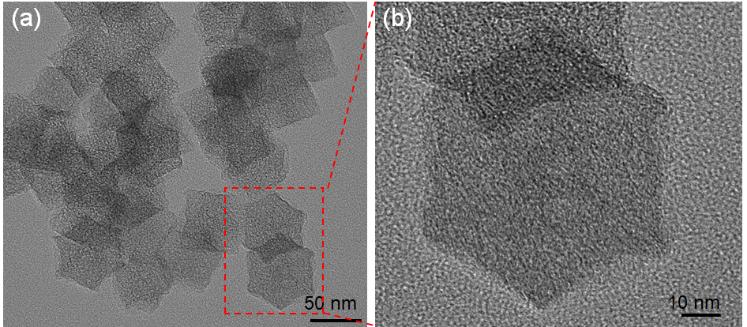


**Figure S33.** TEM images of RhB-PEG@ Fe_2_-NCs.


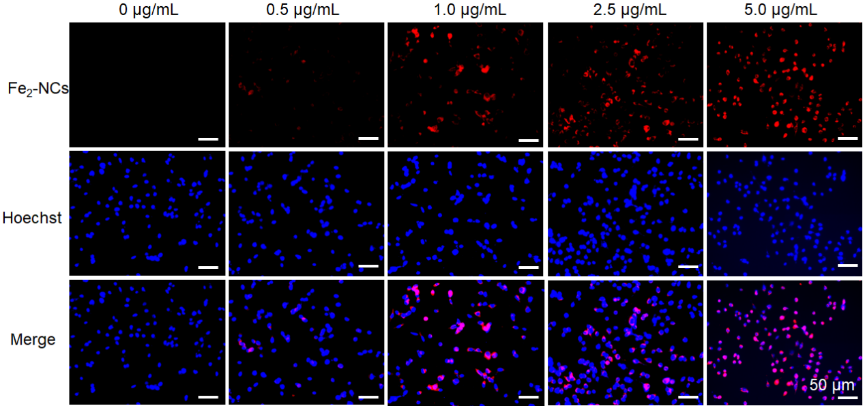


**Figure S34.** Fluorescence images after cellular internalization (1 h) of Fe_2_-NCs at different concentrations.


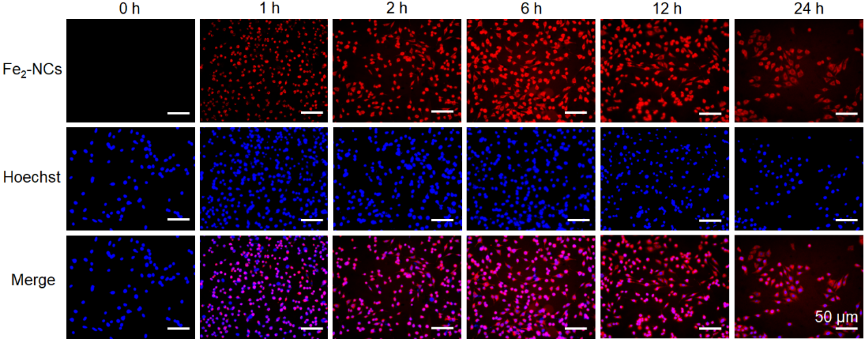


**Figure S35.** Fluorescence images after cellular internalization of Fe_2_-NCs (2.5 µg/mL) at different lengths of time.

**
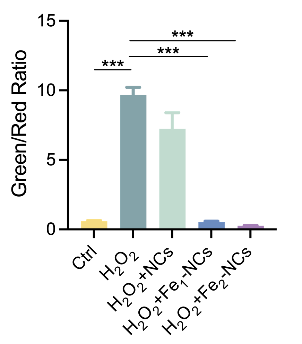
**

**Figure S36.** Quantitative analysis of the green/red fluorescence ratio (n = 3).


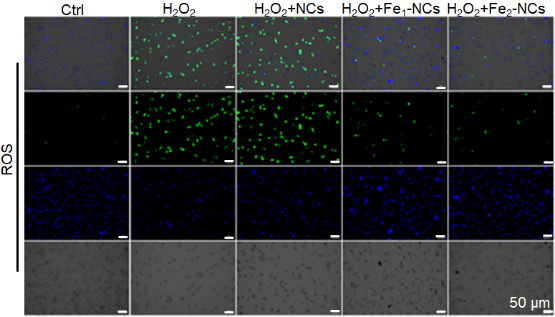


**Figure S37.** Intracellular ROS visualization via Hoechst 33342 fluorescence under oxidative stress modulation (Scale bar: 50 μm, n=3).


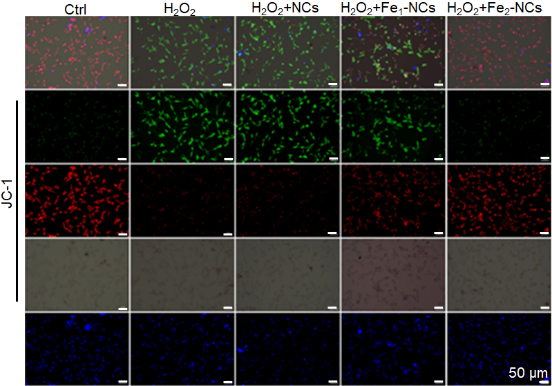


**Figure S38.** The mitochondrial polarization status by detecting mitochondrial membrane potential using JC-1 probe (Scale bar: 50 μm, n=3).


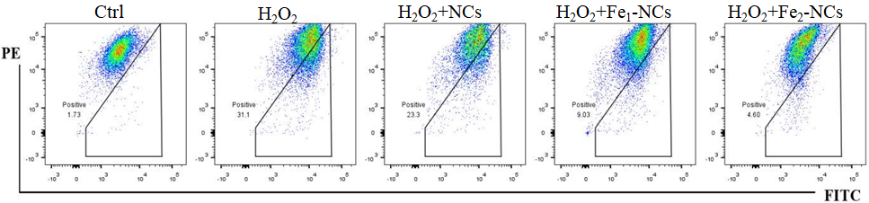


**Figure S39.** Flow cytometry analysis of mitochondrial membrane potential (ΔΨm) in H_2_O_2_-induced cells treated with the prepared groups (Ctrl, H_2_O_2_, H_2_O_2_+Fe_1_-NCs and H_2_O_2_+Fe_2_-NCs groups.)

**
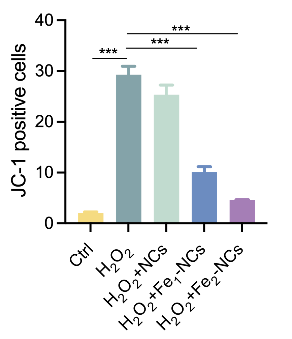
**

**Figure S40.** Quantitative analysis of the red/green fluorescence ratio (n = 3). Data are presented as mean ± SD; statistical significance was determined by one-way ANOVA followed by Tukey’s post hoc test (*p* < 0.05, p < 0.01, *p* < 0.001).


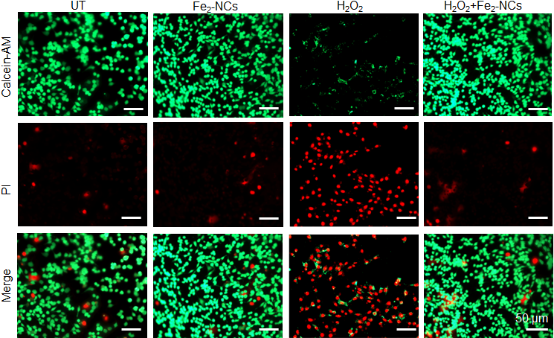


**Figure S41.** Fluorescence images of ATDCs cells after treated with various disposes. The cells were stained by Calcein-AM/PI before the analysis by Fluorescence.


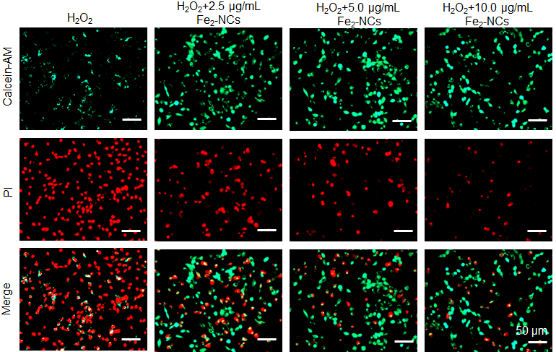


**Figure S42.** Fluorescence images of ATDCs cells after treated at different concentrations. The cells were stained by Calcein-AM/PI before the analysis by Fluorescence.


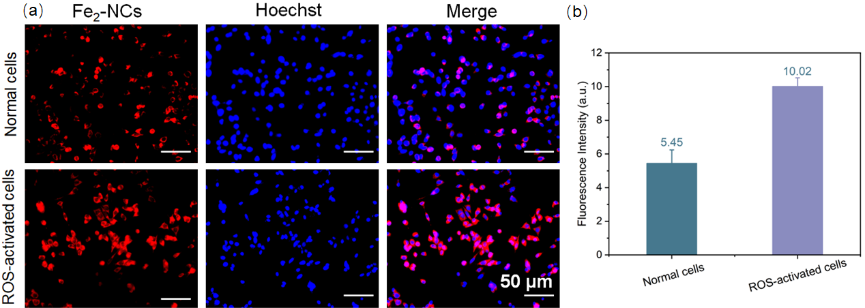


**Figure S43.** (a) Fluorescence images after cellular internalization of Fe_2_-NCs (2.5 µg/mL) within 1 h for normal cells and ROS-activated cells. (b) The corresponding Fluorescence intensity quantified between normal cells and ROS-activated cells**.**


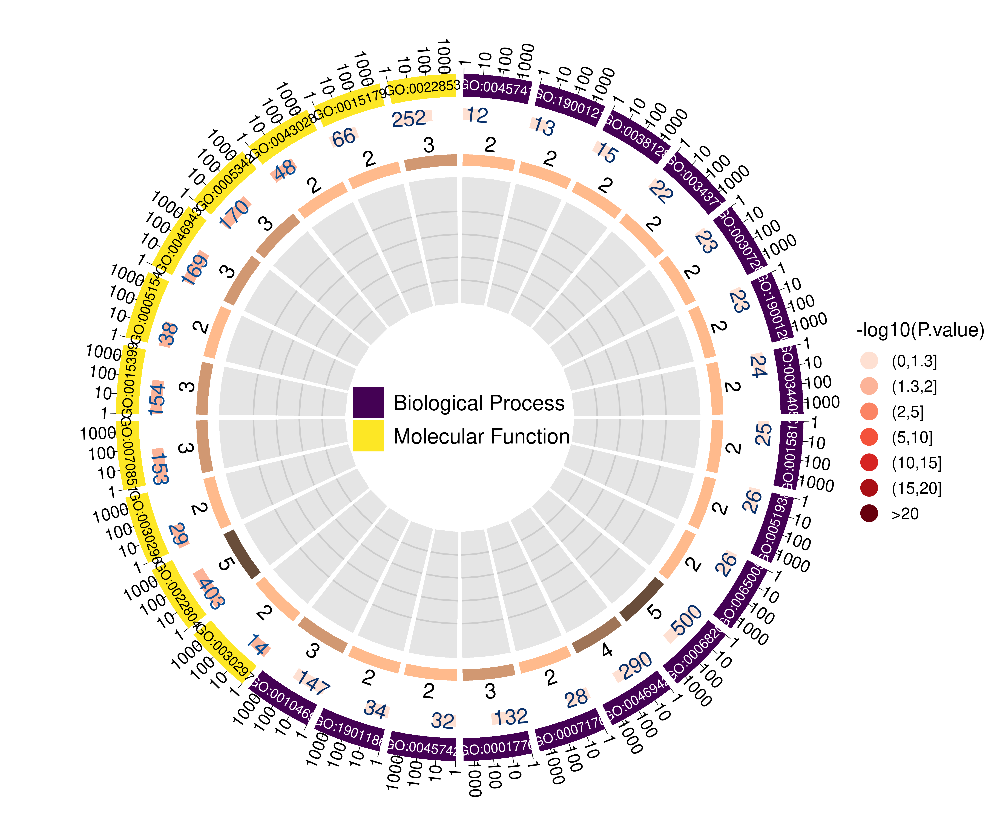


**Figure S44.** GO enrichment analysis of intersection DEGs from biological processes and molecular functions.


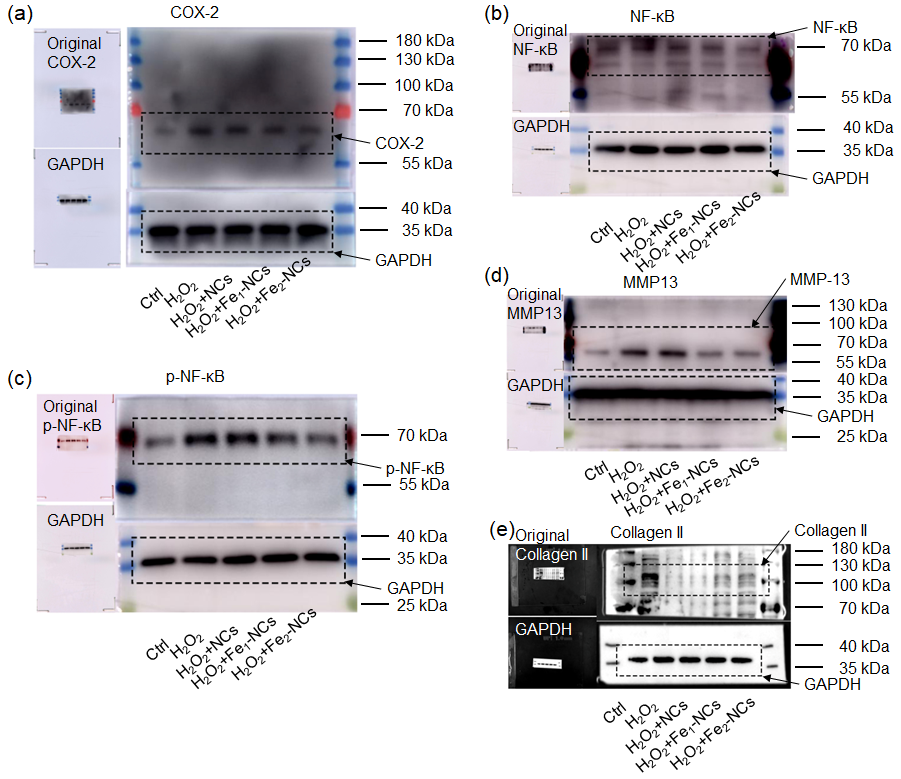


**Figure S45.** The original western blot densitometric analysis of cartilage homeostasis markers: (a) COX-2, (b) NF-κB, (c) p-NF-κB, (d) MMP13 and (e) Collagen II.

**
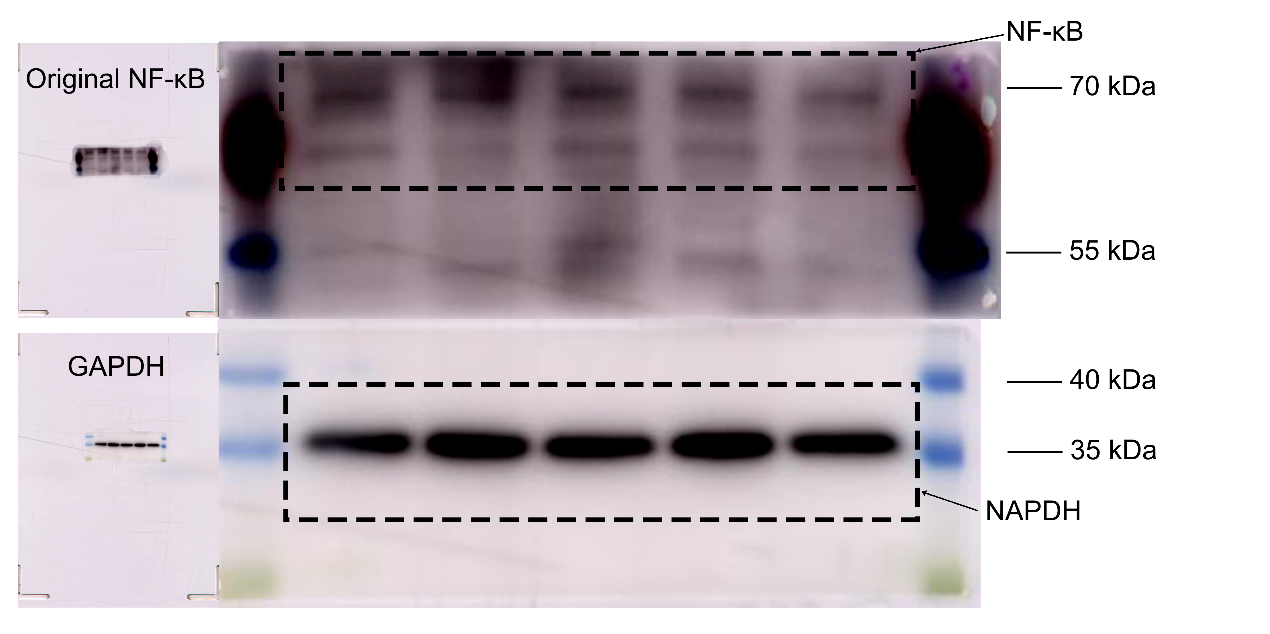
**

**Figure S46.** Western blot densitometric analysis of total NF-κB.


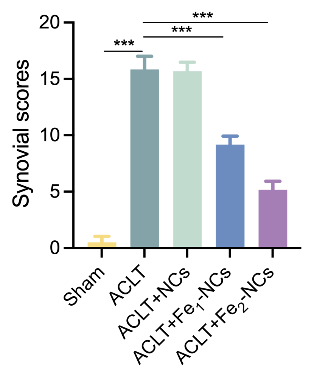


**Figure S47.** Quantitative microarchitectural parameters: Synovival scores.


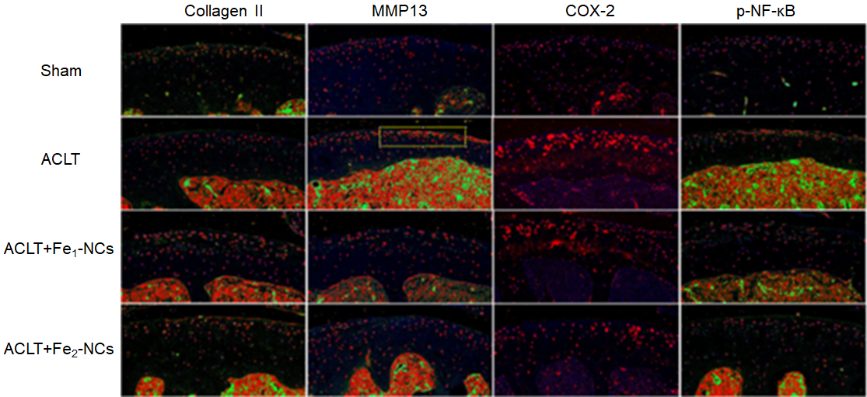


**Figure S48.** Immunofluorescence staining of cartilage extracellular matrix biomarkers in different groups (Sham, ACLT, ACLT+Fe_1_-NCs, and ACLT+Fe_2_-NCs).

**
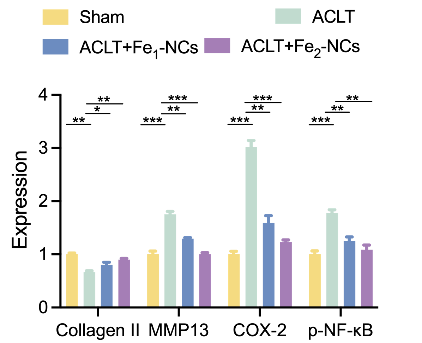
**

**Figure S49.** Quantitative analysis of immunofluorescence staining in cartilage extracellular matrix.


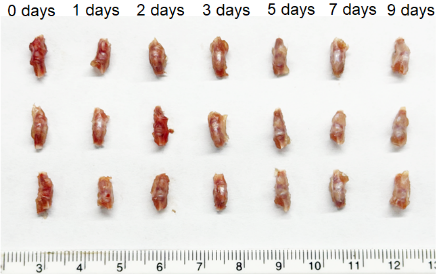


**Figure S50.** Macroscopic photographs of knee joint at different time (0, 1, 2, 3, 5, 7, and 9 days).

**
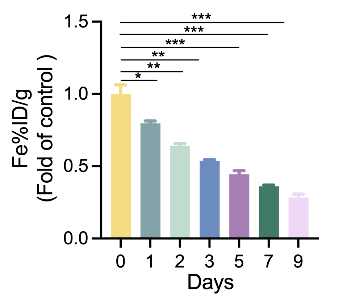
**

**Figure S51.** Macroscopic photographs of knee joint at different time (0, 1, 2, 3, 5, 7, and 9 days).


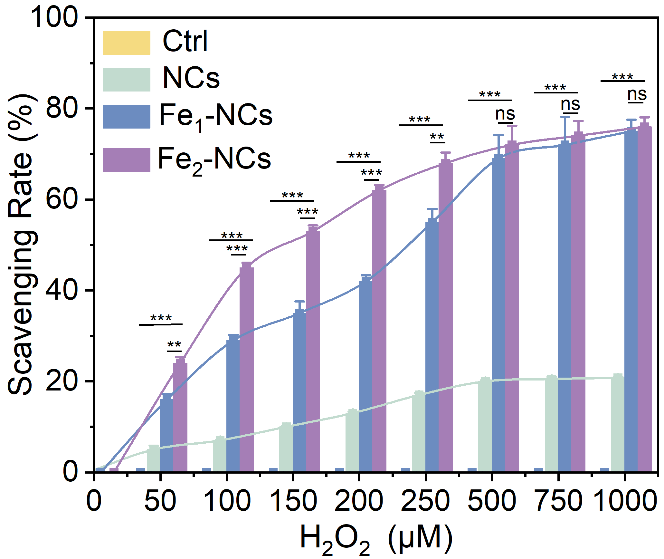


**Figure S52**. The ROS-scavenging activity of Fe_1_-NCs and Fe_2_-NCs across a gradient of H_2_O_2_ concentrations (0, 50, 100, 150, 200, 250, 500, 750 and 1000 μM).


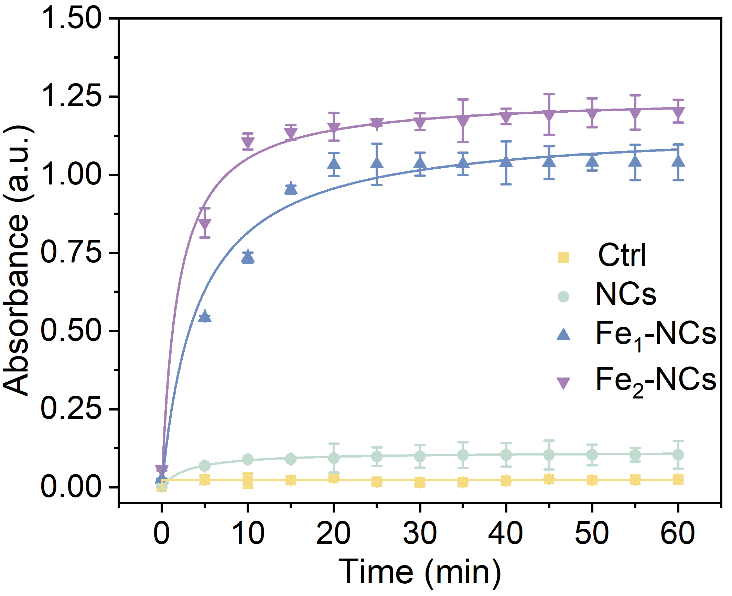


**Figure S53.** Time-resolved kinetic measurements of ROS clearance at a H_2_O_2_ concentration related to *in vitro* experiments (500 μM) over a 0–60 min period.


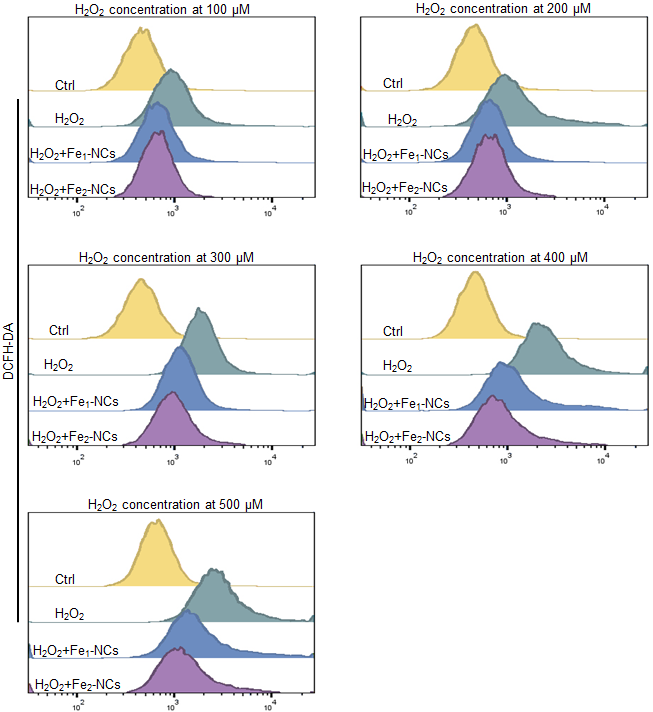


**Figure S54.** Flow cytometry analysis of intracellular ROS levels in chondrocytes treated with Fe_1_-NCs or Fe_2_-NCs under different oxidative stress conditions. Cells were exposed to gradient concentrations of H_2_O_2_ (100–500 μM) and stained with DCFH-DA to monitor ROS levels.

**Note:** Compared with Fe_1_-NCs, Fe_2_-NCs exhibited stronger ROS scavenging capacity, particularly at physiologically relevant concentrations (<500 μM). At higher H_2_O_2_ concentrations (500 μM), the difference between the two nanozymes diminished, likely due to saturation of ROS clearance.


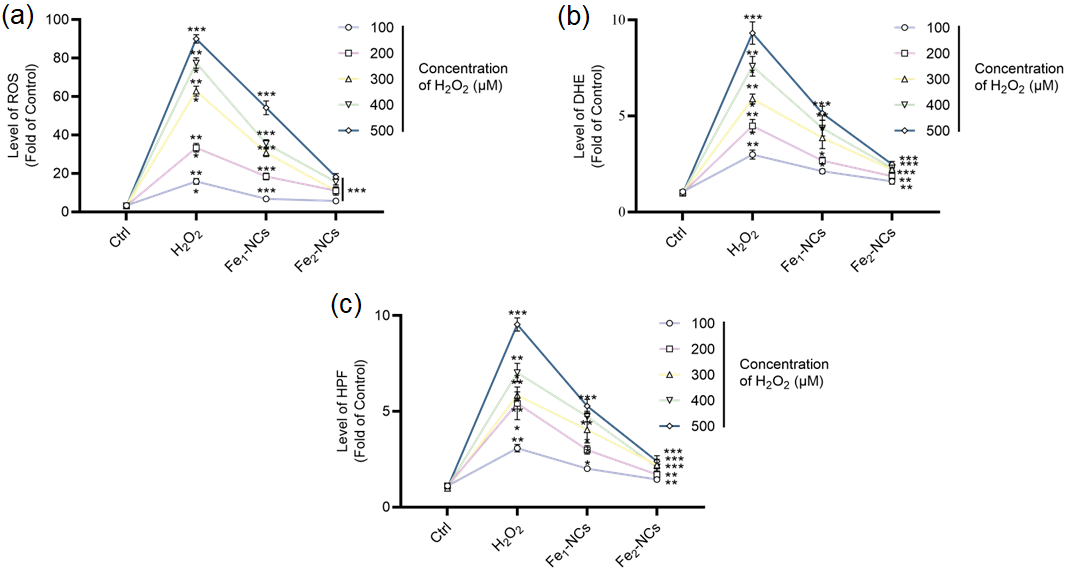


**Figure S55.** Intracellular ROS visualization via ROS species specificity fluorescence under oxidative stress modulation: (a) Total ROS, (b) O_2_•⁻ and (c) •OH.


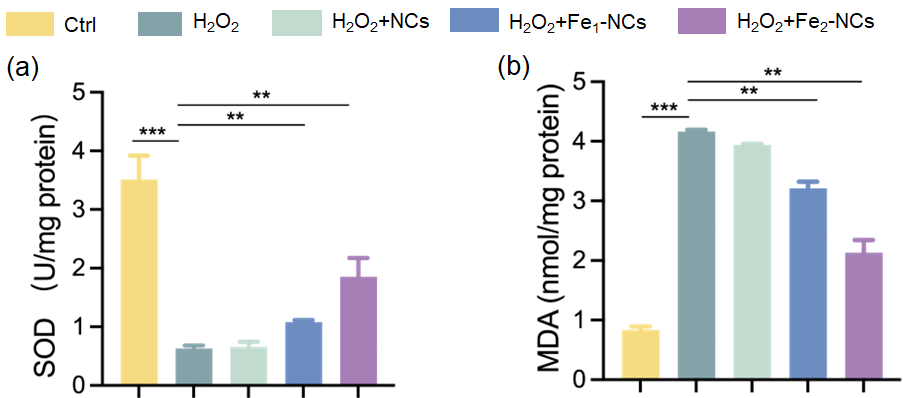


**Figure S56.** (A) SOD-mimetic catalytic kinetics quantification at lower concentrations (2 μg/mL) (n=3). (B) MDA content of cells at lower concentrations (2 μg/mL) with different treatments (n=3).


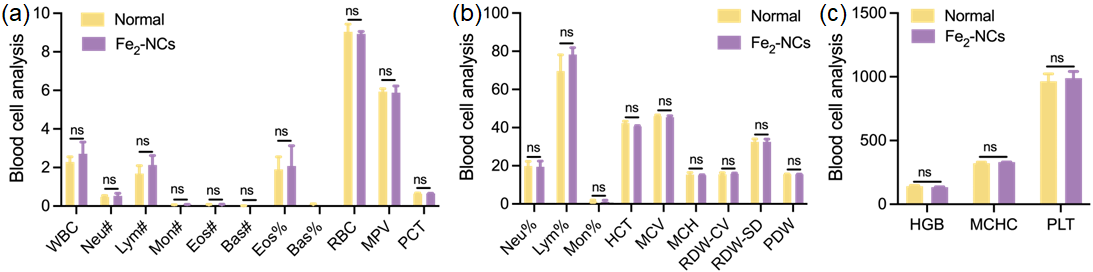


**Figure S57.** Long-term hematological safety evaluation of Fe₂-NCs *in vivo*. Peripheral blood samples were collected from mice after 8 weeks of repeated intra-articular injections and analyzed by complete blood count (CBC). (a) Total and differential leukocyte counts. (b) Erythrocyte indices, as well as hemoglobin-related parameters HGB and MCHC. (c) Platelet-related parameters. All values remained within ±5% of the control group mean, with no statistically significant differences (NS, p > 0.05), indicating good biocompatibility and low systemic hematological toxicity of Fe_2_-NCs *in vivo*.


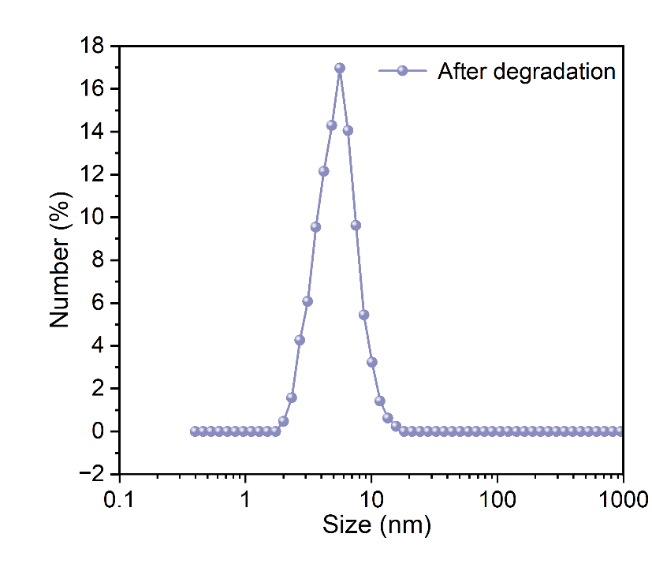


**Figure S58.** Size distribution of the prepared Fe_2_-NCs by DLS after degradation.


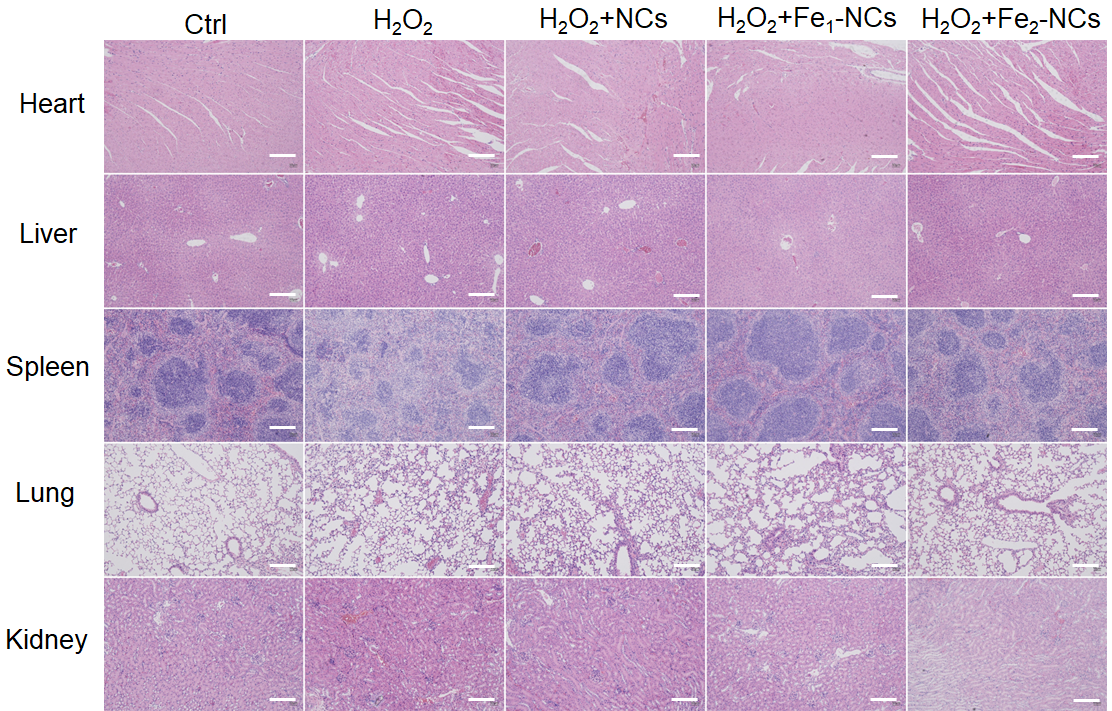


**Figure S59.** Biosafety and toxicity evaluation *in vivo*. Representative images of H&E staining of heart, liver, spleen, lung and kidney of each treatment group after 8 w (Scale bar presents 50 µm)

**Table S1.** The ICP-AES and elemental analysis data of Fe_2_-NCs and Fe_1_-NCs, respectively.

| Samples (wt%) | ^[a]^Fe | ^[b]^C | ^[b]^N |
| --- | --- | --- | --- |
| Fe_1_-NCs | 1.37 | 89.92 | 10.03 |
| Fe_2_-NCs | 1.42 | 88.24 | 11.68 |

Based on ^[a]^ICP-AES and ^[b]^elemental analysis results.

**Table S2**. EXAFS Fitting parameters at the Fe k-edge for Fe_2_-NCs and Fe_1_-NCs.

| Sample | Shell | *CN^a^* | *R*(Å)*^b^* | *σ*^2^(Å^2^)*^c^* | Δ*E*_0_(eV)*^d^* | *R* factor |
| --- | --- | --- | --- | --- | --- | --- |
| Fe-foil | Fe-Fe | 8* | 2.469±0.011 | 0.0050±0.0011 | 6.7±1.9 | 0.0062 |
|  | Fe-Fe | 6* | 2.846±0.018 | 0.0059±0.0020 | 6.0±3.1 |  |
| FePc | Fe-N | 4.0±0.3 | 1.970±0.001 | 0.0061±0.0016 | 0.6±0.5 | 0.0093 |
| Fe_1_-NCs | Fe-N | 4.1±0.2 | 2.020±0.001 | 0.009±0.0035 | 0.5±0.1 | 0.0060 |
| Fe_2_-NCs | Fe-N | 4.1±0.6 | 2.027±0.001 | 0.0078±0.0027 | 2.1±0.6 | 0.0106 |
|  | Fe-Fe | 1.2±0.3 | 2.496±0.001 | 0.0022±0.0021 | -1.6±1.1 |  |

*^a^CN*, coordination number; *^b^R*, the distance to the neighboring atom; *^c^σ*^2^, the Mean Square Relative Displacement (MSRD); *^d^ΔE*_0_, inner potential correction; *R* factor indicates the goodness of the fit. *S*0^2^ was fixed to 0.742, according to the experimental EXAFS fit of Fe foil by fixing *CN* as the known crystallographic value. * This value was fixed during EXAFS fitting, based on the known structure of Fe. Fitting range: 3.0 ≤ *k* (/Å) ≤ 12.0 and 1.0 ≤ *R* (Å) ≤ 3.0 (Fe foil); 2.0 ≤ *k* (/Å) ≤ 11.0 and 1.0 ≤ *R* (Å) ≤ 2.0 (FePc); 3.0 ≤ *k* (/Å) ≤ 12.0 and 1.0 ≤ *R* (Å) ≤ 2.9 (Fe_1_-NCs); 2.5 ≤ *k* (/Å) ≤ 12.0 and 1.0 ≤ *R* (Å) ≤ 2.8 (Fe_2_-NCs). A reasonable range of EXAFS fitting parameters: 0.700 < *Ѕ*_0_^2^ < 1.000; *CN >* 0; *σ*^2^ > 0 Å^2^; |Δ*E*_0_| < 15 eV; *R* factor < 0.02.

**Table S3.** The content of N species in Fe_2_-NCs and Fe_1_-NCs, respectively.

| Samples | Pyridinic N | Fe-N | Pyrrolic N | Graphitic N | Oxidized N |
| --- | --- | --- | --- | --- | --- |
| Fe_1_-NCs | 2.93 | 2.87 | 3.05 | 1.02 | 0.16 |
| Fe_2_-NCs | 2.95 | 2.64 | 3.56 | 2.18 | 0.35 |

**Table S4.** Comparison of CAT-Like activity in apparent Michaelis-Menten constant (K_M_) and maximum reaction rate (V_max_) between Fe_2_-NCs and Fe_1_-NCs.

| Samples | Substrate | K_M_ (M) | V_max_ (M/min) |
| --- | --- | --- | --- |
| Fe_2_-NCs  (20 µg/mL) | H_2_O_2_ | 0.1631 | 5.378 |
| Fe_1_-NCs  (20 µg/mL) | H_2_O_2_ | 0.2958 | 3.095 |
| Natural CAT  (20 µg/mL) | H_2_O_2_ | 0.3473 | 2.037 |

**Table S5.** Comparison of OXD-Like activity in apparent Michaelis-Menten constant (K_M_) and maximum reaction rate (V_max_) between Fe_2_-NCs and Fe_1_-NCs.

| Samples | Substrate | K_M_ (M) | V_max_ (M/min) |
| --- | --- | --- | --- |
| Fe_2_-NCs  (20 µg/mL) | TMB | 0.8304 | 1.136 |
| Fe_1_-NCs  (20 µg/mL) | TMB | 1.972 | 0.7103 |

**Table S6.** Primer sequences used in PCR.

| Gene | Forward | Reverse |
| --- | --- | --- |
| Mouse-NFkB1 | GCTGCCAAAGAAGGACACGACA | GGCAGGCTATTGCTCATCACAG |
| Mouse-  NOX4 | CGGGATTTGCTACTGCCTCCAT | GTGACTCCTCAAATGGGCTTCC |
| Mouse-PTGS2 | GCGACATACTCAAGCAGGAGCA | AGTGGTAACCGCTCAGGTGTTG |
